# Synthesis and Application of Trehalose Materials

**DOI:** 10.1021/jacsau.2c00309

**Published:** 2022-07-06

**Authors:** Daniele Vinciguerra, Madeline B. Gelb, Heather D. Maynard

**Affiliations:** † Department of Chemistry and Biochemistry, University of California, Los Angeles, 607 Charles E. Young Drive East, Los Angeles, California 90095-1569, United States; ‡ California NanoSystems Institute, University of California, Los Angeles, 570 Westwood Plaza, Los Angeles, California 90095-1569, United States

**Keywords:** trehalose, polymer, stabilization, hydrogel, protein

## Abstract

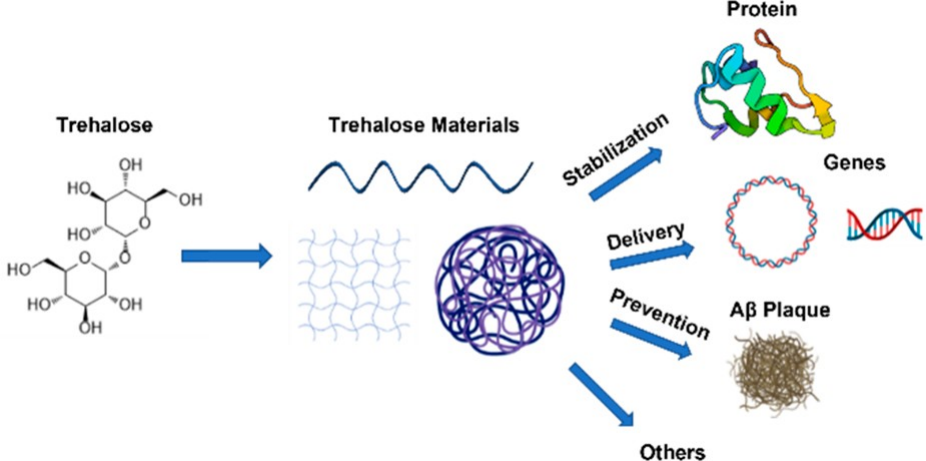

Trehalose is a naturally occurring, nonreducing disaccharide that is widely used in the biopharmaceutical, food, and cosmetic industries due to its stabilizing and cryoprotective properties. Over the years, scientists have developed methodologies to synthesize linear polymers with trehalose units either in the polymer backbone or as pendant groups. These macromolecules provide unique properties and characteristics, which often outperform trehalose itself. Additionally, numerous reports have focused on the synthesis and formulation of materials based on trehalose, such as nanoparticles, hydrogels, and thermoset networks. Among many applications, these polymers and materials have been used as protein stabilizers, as gene delivery systems, and to prevent amyloid aggregate formation. In this Perspective, recent developments in the synthesis and application of trehalose-based linear polymers, hydrogels, and nanomaterials are discussed, with a focus on utilization in the biomedical field.

## 1 Introduction

**Figure 1:**
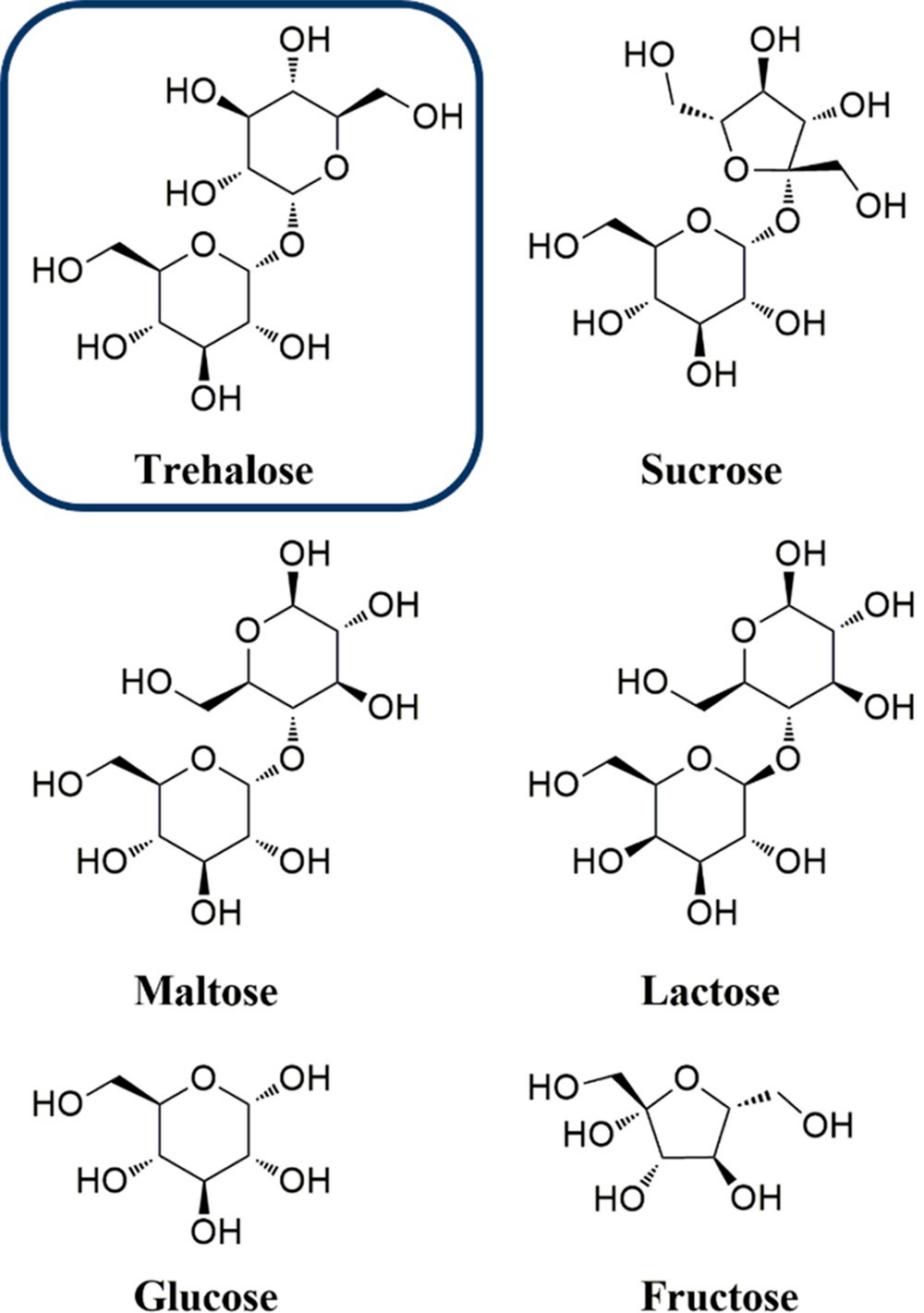
Structures of trehalose and other common sugars.

Trehalose is a naturally occurring, nonreducing disaccharide formed by the α,α-1,1 glycosidic linkage of two glucose units (α-d-glucopyranosyl-α-d-glucopyranoside) (Figure 1). This specific bond bends trehalose into a rigid clamshell structure.^1,2^ Trehalose, as an amorphous sugar, has the highest glass transition temperature (*T*_g_) of disaccharides at 114 °C^3^ and an anhydrous melting temperature (*T*_m_) of 203 °C.^4^ The exact stereochemical arrangement of the many hydroxyl groups is important in the formation of specific hydrogen bonds.^1,2,5,6^ It is well established that trehalose acts as a bioprotective agent against various environmental stresses such as freezing and drying, and is produced as an osmoprotectant by some microorganisms and plants in response to stress; although, it is not naturally found in mammals. Trehalose is often more effective than other sugars at maintaining cellular integrity by protecting the native three-dimensional structure of cell bilayers and proteins, inhibiting their denaturation, degradation, and aggregation.^3,4,7−11^ Relative to other sugars, trehalose has a higher affinity for water and occupies a larger volume when hydrated.^10,11^ This property, however, is also likely responsible for the relatively high viscosity of trehalose solutions.^10^ This drawback is often accepted in the medical field in favor of the better stabilization properties and relative inertness of trehalose, which lacks the free aldehyde groups susceptible to unwanted Maillard reactions that are common with other sugars.^4,9,12^ Furthermore, as the glycosidic bond is highly stable, trehalose is less susceptible to hydrolysis, thereby making it more inert than sucrose, the other common nonreducing sugar (Figure 1). Nonetheless, when ingested, the trehalose glycosidic bond is hydrolyzed in humans by the intestinal enzyme trehalase to form two molecules of glucose, which are subsequently adsorbed and metabolized.^13^

**Figure 2:**
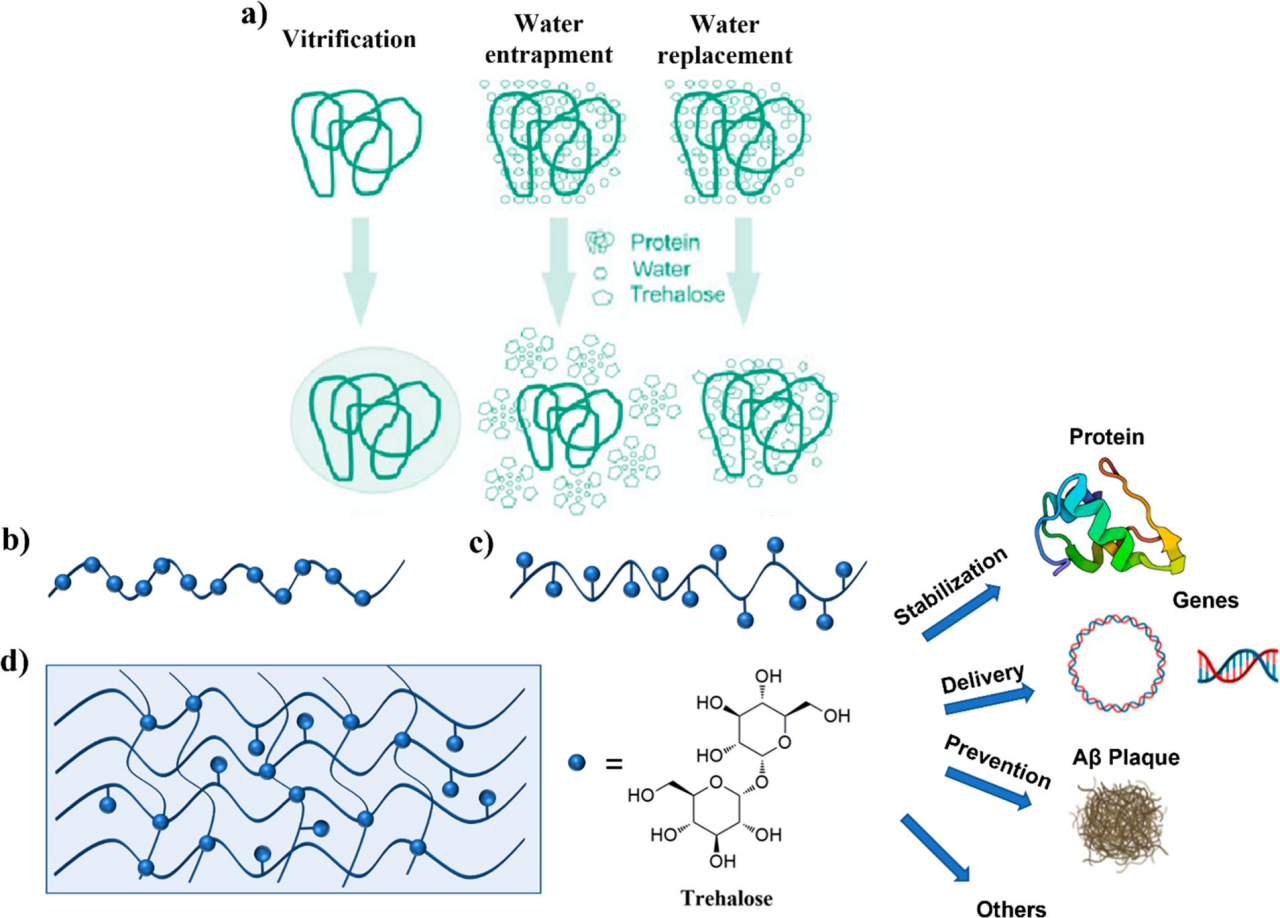
Schematic representation of (a) trehalose proposed stabilization mechanisms. Adapted with permission from ref (4). Copyright 2009 Wiley. Schematic representation of (b) polymer with trehalose in the backbone, (c) polymer carrying trehalose in the side chains, and (d) thermoset or hydrogel network with trehalose as cross-linker or in the side chain and examples of their applications.

Trehalose is a highly versatile stabilizer that has already been implemented in the biomedical field in a wide variety of formulations. Despite its widespread use, the precise stabilization mechanism is still disputed and most likely depends in part on the environmental conditions^4^ and the type of molecule being stabilized.^7^ The multiple theories to explain trehalose stabilization include water entrapment or preferential exclusion, water replacement, and vitrification (Figure 2a).^4^ Generally, these three mechanisms rely on trehalose decreasing the local mobility of biomacromolecules by sequestering or replacing water, or by resisting solvent crystallization through the formation of a glassy matrix around unstable biomolecules, respectvely.^6,8,10,11^ The difference between the water entrapment and replacement theories lies in whether a solvation layer around the protein is present or if trehalose is directly interacting with the protein surface. Vitrification requires trehalose to form an amorphous or glassy matrix to prevent the formation of ice crystals that cause “freeze concentration”, that, is the concentration of solute in the remaining liquid which can result in protein denaturation.^12^

Despite early research seeking a single answer to the stabilization mechanism question, it is possible that multiple mechanisms work simultaneously and/or are influenced by the specific biomacromolecule. Repeated lyophilization studies indicated that trehalose must continue to maintain direct or indirect hydrogen bonds with the polar residues of proteins to maintain the native conformations of the biomolecules once dried.^3,4,12^ One of the earliest molecular dynamics (MD) studies on trehalose concluded that trehalose does not affect the structure of water in sufficiently dilute conditions and therefore trehalose must stabilize proteins by water replacement.^5^ Only a few years later, further MD exploration contradicted this theory, finding that trehalose has significant water interactions and disrupts the natural tetrahedral network of water, attributing the high degree of order in part to the conformational rigidity of trehalose.^1,2,14^ Additionally, the authors concluded that, while trehalose clearly had kosmotropic effects and could cause water entrapment, the ability to structure water does not exclude water replacement or vitrification as the mechanism by which trehalose stabilizes biomolecules. This view has been demonstrated experimentally with spectroscopic experiments reporting the importance of water entrapment and destructuring,^14−16^ water replacement,^8,17^ and the formation of a glassy matrix by trehalose.^3,12,18^ More generally, researchers agree that these mechanisms can and do act in combination to prevent the unfolding, misfolding, and aggregation of biomacromolecules.^1,4^

Regardless of the mechanism, researchers have repeatedly shown trehalose to be a more effective stabilizer than other sugars.^3,10,17^ For instance, in stabilizing pyrophosphatase and glucose 6-phosphate dehydrogenase against heat, trehalose was about twice more effective than the same mole concentration of sucrose and maltose or double the mole concentration of glucose or fructose.^10^ Comparatively, trehalose was a better liposome protectant than sucrose against lyophilization followed by storage or heating conditions.^3^

As a small molecule, trehalose is a highly effective stabilizer and has been incorporated into polymers and other polymeric materials for even more dramatic stabilization results.^19−23^ As our group has previously shown, in heat and lyophilization stability assays, proteins retain greater bioactivity in the presence of trehalose polymers (excipient, conjugate, hydrogel, or nanogel) than alone or with the same weight concentration of trehalose.^19,20,24^ In the same vein, trehalose nanoparticles were better than trehalose alone at preventing proteins from undergoing fibrillation.^21^ Cryopreservation assays of different mammalian cells in the presence of increasing amounts of trehalose polymer similarly showed improved cell growth after freezing with a polymer rather than trehalose alone.^22^ These studies have also shown that trehalose materials stabilize biomolecules and cells at a lower relative concentration than that of trehalose itself.^21,22,25^ Unexpectedly, with this improved stabilization work, linear trehalose polymers did not show similarly high viscosities as the small molecule or more complex fluid flow properties.^26^

Taken altogether, these characteristics firmly demonstrate the utility of trehalose polymers in the rapidly growing biopharmaceutical market. In this Perspective, different polymerization strategies to prepare trehalose-based polymers and materials will be first discussed and critically analyzed. Afterward, various applications in the biomedical field, such as protein stabilization, gene delivery, and amyloid aggregate prevention, will be presented and discussed (Figure 2b–d). To conclude, we will provide an outlook and propose future directions for the field to move forward. Trehalose glycolipids and their polymeric derivatives^27^ will not be discussed as these have already been reviewed elsewhere.^28,29^

## 2 Polymerization Strategies

**Figure 3:**
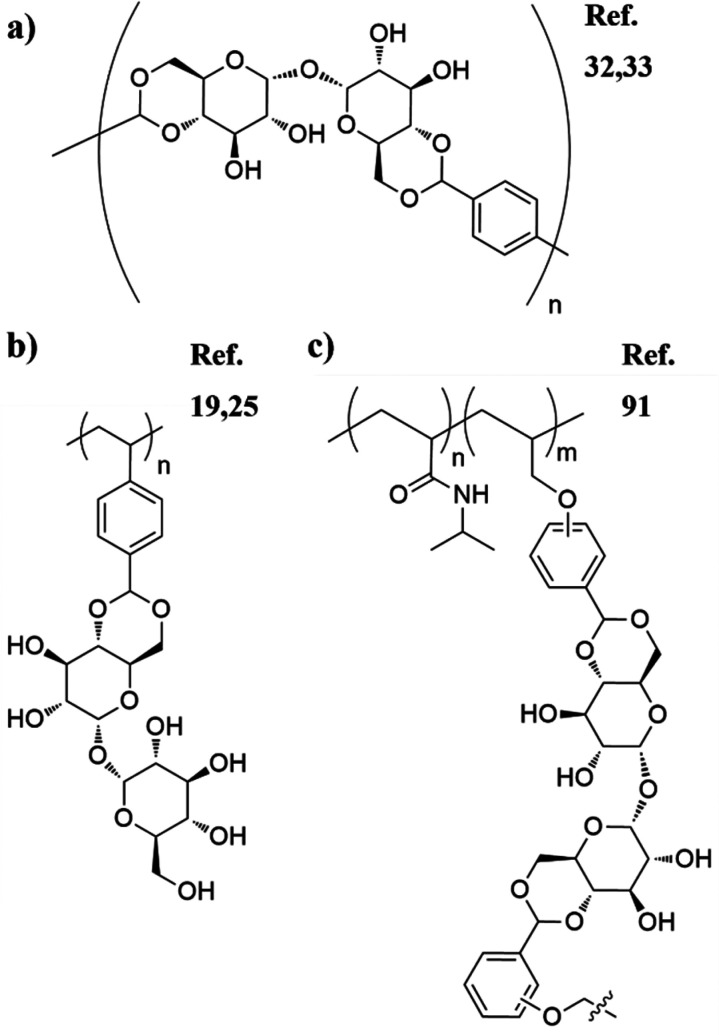
Representative selection of a (a) linear polymer with trehalose in the backbone, (b) linear polymer with trehalose on the side chain, and (c) hydrogel with trehalose as cross-linker.

Many strategies have been employed over the years to synthesize poly(trehalose) polymers with various architectures. Linear polymers can be prepared following two different approaches: (a) step-growth polymerization where trehalose is incorporated by polyaddition or polycondensation directly into the backbone of the polymer or (b) chain-growth polymerization where trehalose is linked to unsaturated monomers as side chains and, typically, radical polymerization affords linear chains with trehalose pendant on the side chains. Cross-linked materials can be prepared by (c) curing of trehalose containing multiple olefins to afford insoluble thermoset resins or cross-linking of poly(trehalose) in aqueous conditions to give hydrogels (Figure 3).

### 2.1 Backbone Trehalose

**Figure 4:**
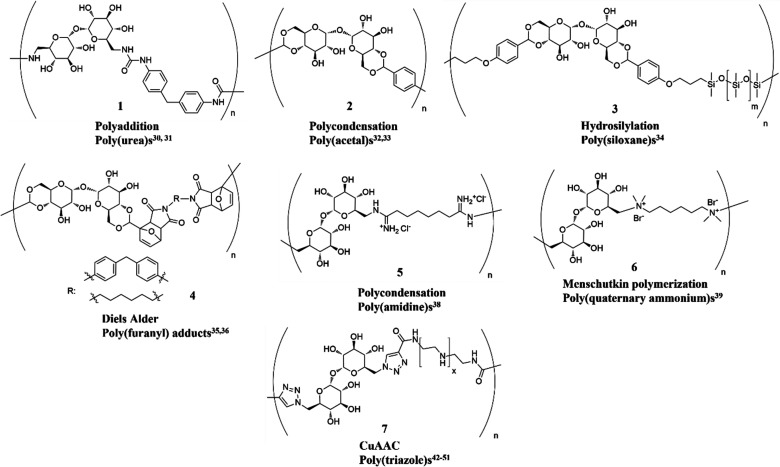
Representative selection of polymers and reaction classes with trehalose in the backbone.

A selection of polymers with trehalose in the backbone is summarized in Figure 4. The first attempt to prepare linear trehalose polymers was reported in 1979 by Kurita et al., when the authors employed direct addition polymerization to copolymerize trehalose with diisocyanates yielding polyurethanes **1**, although likely producing branched polymers as a side product.^30^ They later resolved this issue by synthesizing diaminotrehalose using sequential protection–deprotection steps to selectively modify the C-6,6’ hydroxyl groups. The modified trehalose was then reacted with various diisocyanates, such as diphenylmethane diisocyanate, to afford polyureas by polyaddition using various polar solvents at temperatures ranging from 5 to 20 °C. The resulting polymers could be biodegraded using trehalase or α-amylase.^31^ Similarly, trehalose hydroxyls could be reacted with terephthalaldehyde, terephthalaldehyde bis(dimethyl acetal), or 1,*x*-bis(2-formylphenoxy)alkanes (*x* = 4–12) to afford polyacetals **2** by polycondensation.^32,33^ This strategy possessed the advantage of being regioselective for C-6,6’ hydroxyls with no protection steps required. However, this methodology presented some clear disadvantages such as harsh polymerization conditions, low (8.5 kDa) maximum molecular weight (MW) obtained, no glass transition temperature (*T*_g_) found up to the decomposition temperature (*T*_d_) of 325 °C,^32^ and the formation of a mixture of polymers with different end groups or even cyclization.^33^ To overcome these drawbacks, Teramoto et al. designed a different strategy to regioselectively modify trehalose with 4-allyl-oxybenzaldehyde and then polymerize by hydrosilylation with SiH-terminated dimethylsiloxane oligomers.^34^ Polymers **3** with a MW up to 50 kDa were obtained when the mixture was heated at 80 °C for 72 h. Yields were generally high (ca. 80%), but MW polydispersity (*Đ*) was also very high, averaging 3.5. The polymers presented two *T*_g_ peaks: one (ca. −110 °C) independent of and one (96–152 °C) dependent on siloxane oligomer segment length.^34^ In parallel, they developed a synthetic strategy to afford degradable linear poly(trehalose) **4** by exploiting a Diels–Alder reaction between trehalose bearing difurfurylidene and bismaleimides. At a high temperature (140 °C), the polymer underwent a retro Diels–Alder and degraded into its monomers.^35^ In a follow up study, the two strategies were combined by using difurfurylidene trehalose and maleimide bearing dimethylsiloxanes oligomers. The degradable and flexible poly(trehalose-siloxanes) presented similar advantageous thermal properties to the previous siloxane copolymer while retaining degradability from the Diels–Alder reversibility.^36^ Finally, trehalose was derivatized to afford a diepoxide and polymerized following the addition of aliphatic diamines in the presence of a base catalyst. While the trehalose diepoxide had low solubility in various organic solvents, requiring the polymerization to be conducted in 1-methyl-2-pyrrolidone at 200 °C, the resulting polymer was soluble in a range of organic solvents and showed a *T*_g_ of 100 °C and a *T*_d_ of 320 °C.^37^

Another early development in the polycondensation approach involved reacting monomers containing amine-reactive imidoester groups with diaminotrehalose in the presence of sodium carbonate to afford cationic polyamidines **5**. The polymerization only took 16 h, but yields were limited (23–45%).^38^ As part of the development of a family of sugar-containing polycations, trehalose was functionalized in the 6 and 6’ positions with dimethylamine, which proceeded through a diiodide intermediate. The tertiary amines were then reacted with 1,6-dibromohexane via Menschutkin reaction, yielding polycations **6** bearing quaternary amines. The polymerization was conducted at 40 °C for 3 days, but yields were similarly modest (32–38%).^39^

The above-mentioned strategies share some common advantages, such as resulting in copolymers with well-defined monomer sequences and often bearing a degradable backbone. Nonetheless, some disadvantages are also noticeable. Each polymerization reaction was limited in monomer scope, as they need to be chemically compatible and often require harsh conditions. Moreover, polymers were generally obtained in low yield and conversion, the polymerizations were difficult to control, resulting in branching or cyclization, and MWs were often low with high polydispersity.

**Figure 5:**
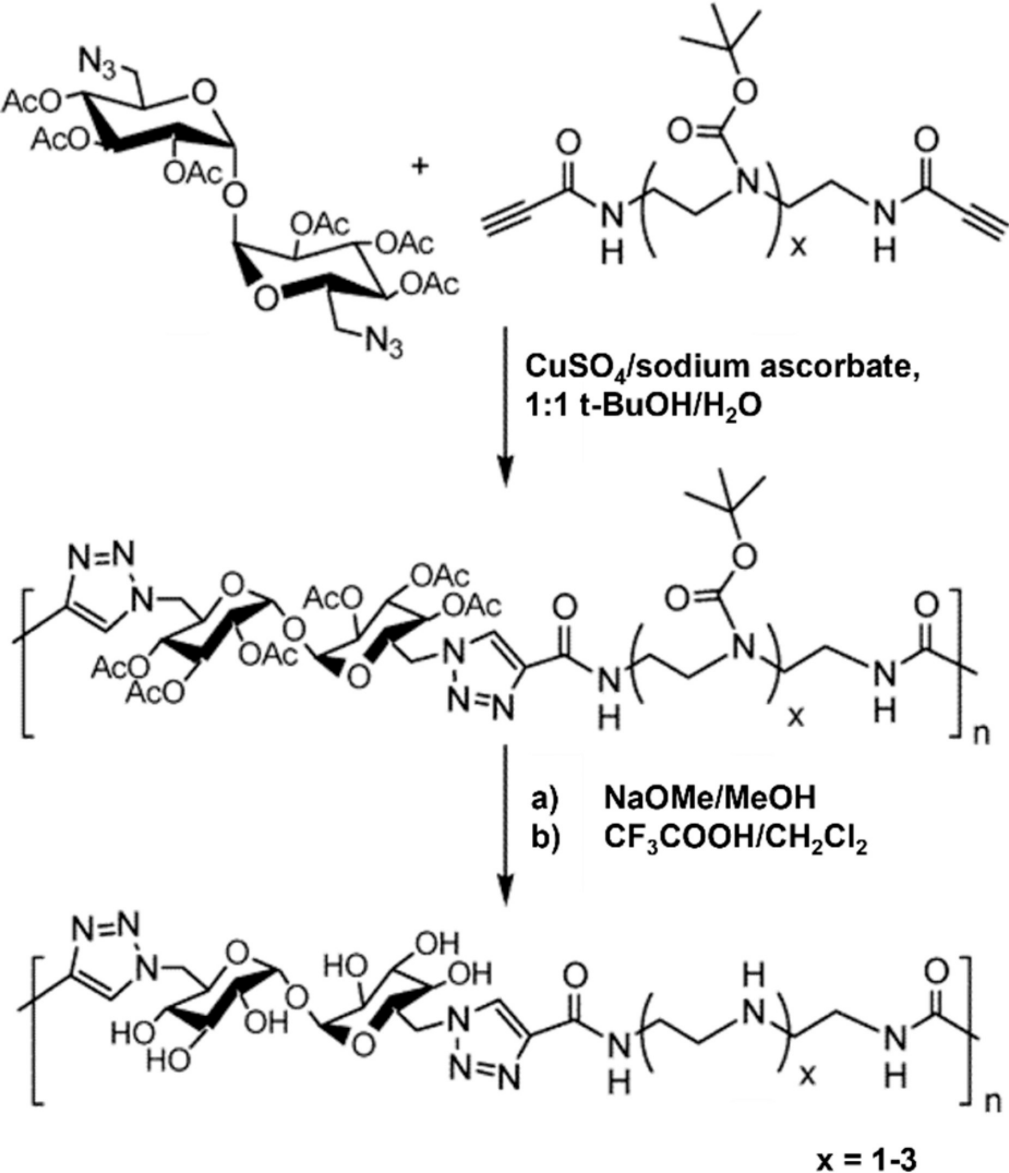
Schematic representation of click polymerization and polymer deprotection. Adapted from ref (42). Copyright 2006 American Chemical Society.

Arguably, Cu^I^-catalyzed azide–alkyne cycloaddition (CuAAC) has been the most successful reaction employed to synthesize polymers with trehalose in the backbone. It solves many of the issues discussed above, requiring friendlier conditions and resulting in higher yields, higher MW, and lower MW dispersities. CuAAC is widely applied both in polymer^40^ and carbohydrate^41^ syntheses. The strategy was popularized by Reineke and co-workers in their effort to synthesize cationic trehalose copolymers for gene delivery (see below for a description of this application).^42−51^ Trehalose bearing two azido groups in the 6 and 6’ positions was prepared by iodination of the respective hydroxyl group, followed by substitution with sodium azide, and finally protection of the remaining hydroxyls with acetyl groups. The diazidotrehalose monomer was polymerized by a reaction with dialkyne-oligoamine monomers. Specifically, an equimolar mixture of the monomers was heated at 50 °C in a 1:1 cosolvent system of *tert*-butyl alcohol and water in the presence of Cu^II^ and sodium ascorbate and stirred for 4–24 h depending on the amine. Finally, the hydroxyls and the amines were deprotected following conventional methods to afford the desired water-soluble copolymer **7** (Figure 5). In addition to the milder polymerization conditions than those for previously discussed strategies, these reactions could easily obtain polymers with higher MW, up to 40 kDa, *Đ* as low as 1.2, and higher degree of polymerization (DP: 56–61), although protection/deprotection steps were still required and removal of the Cu catalyst might be laborious.^42^ This synthetic strategy and these conditions allowed the facile customization of many characteristics of the final polymer including polymer length,^43,46^ amine number,^44,46^ and end group chemistry by introducing a capping monomer at the end of the polymerization.^45,46^ Additionally, a third comonomer could be added, for instance, to add a lanthanide chelating moiety for theranostic purposes.^49^

**Figure 6:**
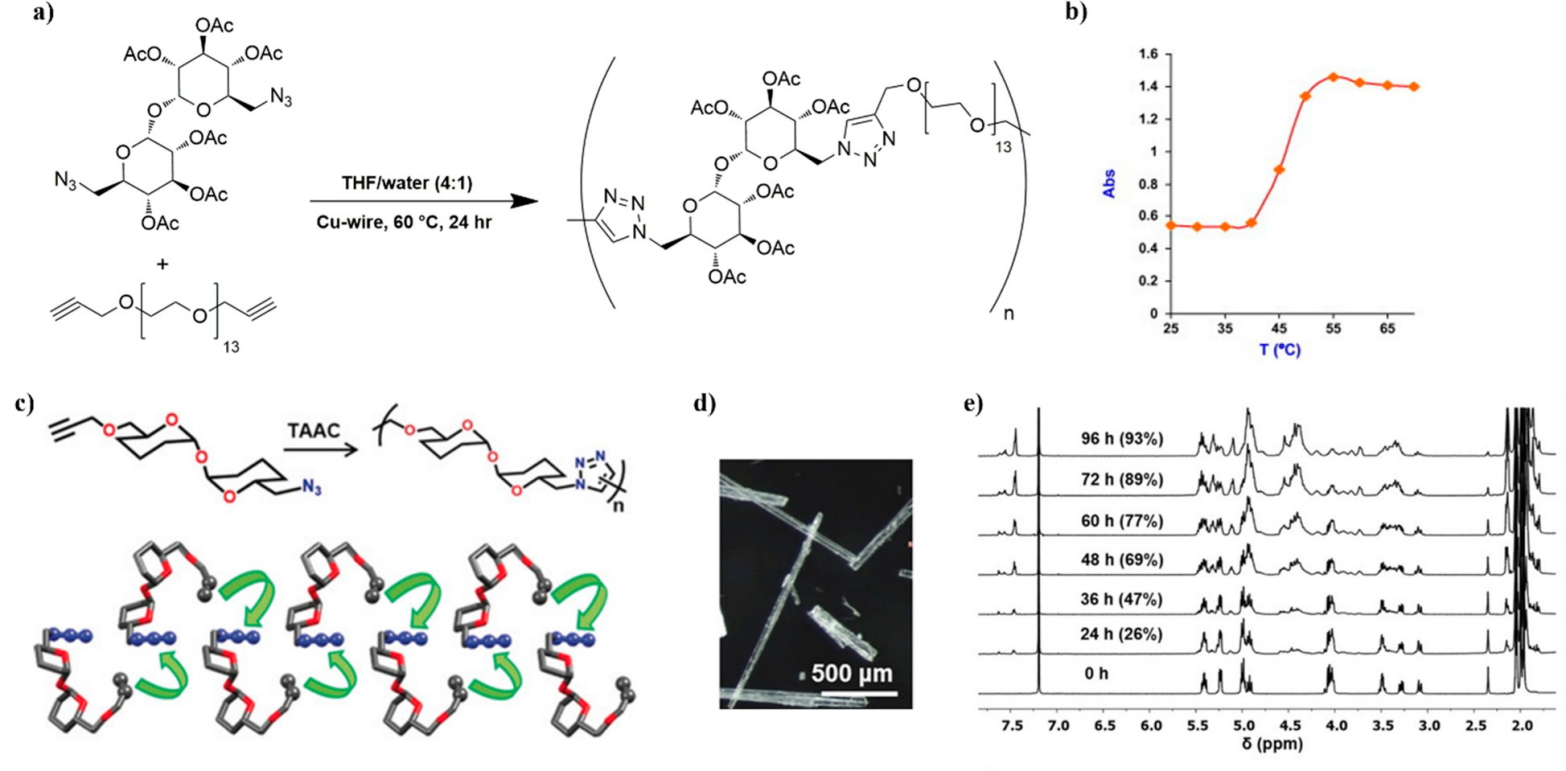
(a) Polymerization scheme to obtain a thermoresponsive trehalose-PEG copolymer by CuAAC, (b) cloud point measurements of an aqueous solution of trehalose-PEG copolymer. Adapted from ref (52) with permission. Copyright 2011 Elsevier. (c) Schematic representation of topochemical azide–alkyne cycloaddition (TAAC) of a trehalose-based monomer. (d) Photographs of the crystals obtained from ethyl acetate/*n*-hexane. (e) Time-dependent ^1^H NMR (CDCl_3_) showing a TAAC reaction in the crystals at 90 °C. Reprinted from ref (53) with permission. Copyright 2020 Wiley.

Other applications involving polymers prepared by CuAAC include a glycopolymer with thermoresponsivity around body temperature^52^ and an asymmetric trehalose bearing both an alkyne and azide for copper-free topochemical azide–alkyne cycloaddition.^53^ In the first case, trehalose primary and secondary alcohols were selectively tosylated and acetylated, respectively. After the initial protection, the tosyl groups were displaced with azides. Dialkyne terminated polyethylene glycols (PEGs) with MWs of 200, 600, and 1000 Da were prepared by reaction with propargyl bromide, and the comonomers were polymerized at 60 °C for 24 h with copper wire as a catalyst (Figure 6a). Acetal-protected glycopolymers containing 600 Da PEG showed a cloud point at 2 mg/mL of 39 °C (Figure 6b), but acetyl deprotection led to water-soluble polymers that did not present thermoresponsive behavior. Interestingly, the analogous polymers of 200 and 1000 Da PEG were insoluble in water or presented a phase transition at 90 °C, respectively.^52^ In the last example, an asymmetric acetylated trehalose monomer bearing either an azide or an alkyne at the primary alcohols was synthesized in five steps, with most yields being above 80%. To avoid challenges from conventional glycopolymer synthesis, topochemical click chemistry was used. The monomer was crystallized from a 2:1 mixture of either ethyl acetate or chloroform and *n*-hexane. The crystals were heated at 90 °C, and the polymer was visible by ^1^H NMR after 24 h, reaching full conversion within 96 h, with the highest attained MW being ca. 7 kDa (Figure 6c–e).^53^ This innovative approach requires more exploration in the future, as it solves issues related to purification and metal removal related to conventional CuAAC chemistry. However, the final product was still acetylated and would require a final deprotection step to produce trehalose for various applications, and the obtained MW was relatively low. Additionally, preparing copolymers might be more difficult than polymerization in the solution phase, due to the potentially incompatible crystal structures and alignment or the inability to prepare crystals from an eventual comonomer.

### 2.2 Side-Chain Trehalose

**Figure 7:**
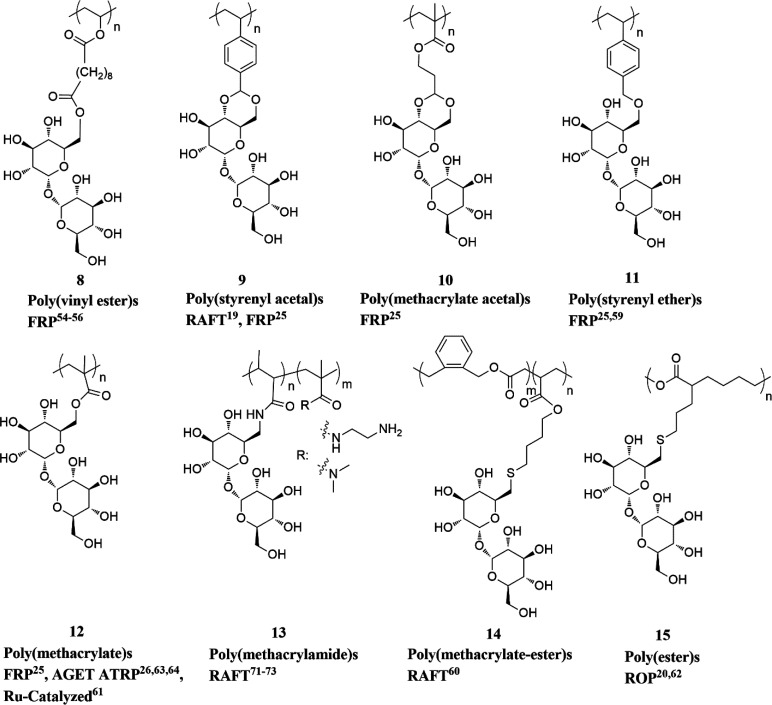
Representative selection of polymers and reaction classes with trehalose in the side chain.

Figure 7 illustrates some examples of polymers bearing trehalose on the side chains. The earliest reports of side-chain trehalose polymers employed enzymes, such as proteases or lipases, to regioselectively modify trehalose at the C6 position with vinyl esters that could subsequently be polymerized by free radical polymerization (FRP) to afford poly(vinyl esters) **8** bearing trehalose on their side chains.^54−56^ The polymers were explored biologically in terms of lectin recognition and enzyme inhibition^55^ or as inhibitors of amyloid β (Aβ) aggregation in Alzheimer’s disease.^56^ In the latter case, to improve amyloid β inhibition, a trehalose acrylamide monomer (TrMA) was prepared in eight steps and polymerized via FRP using 2,2′-azobis(2-amidinopropane)dihydrochloride (AAPD) as the initiator in water. Acrylamide copolymers with different trehalose contents (10, 20, 40, 100 equiv %), constant MW of 100 kDa, and *Đ* values of 1.4–1.6 were prepared.^57^

**Figure 8:**
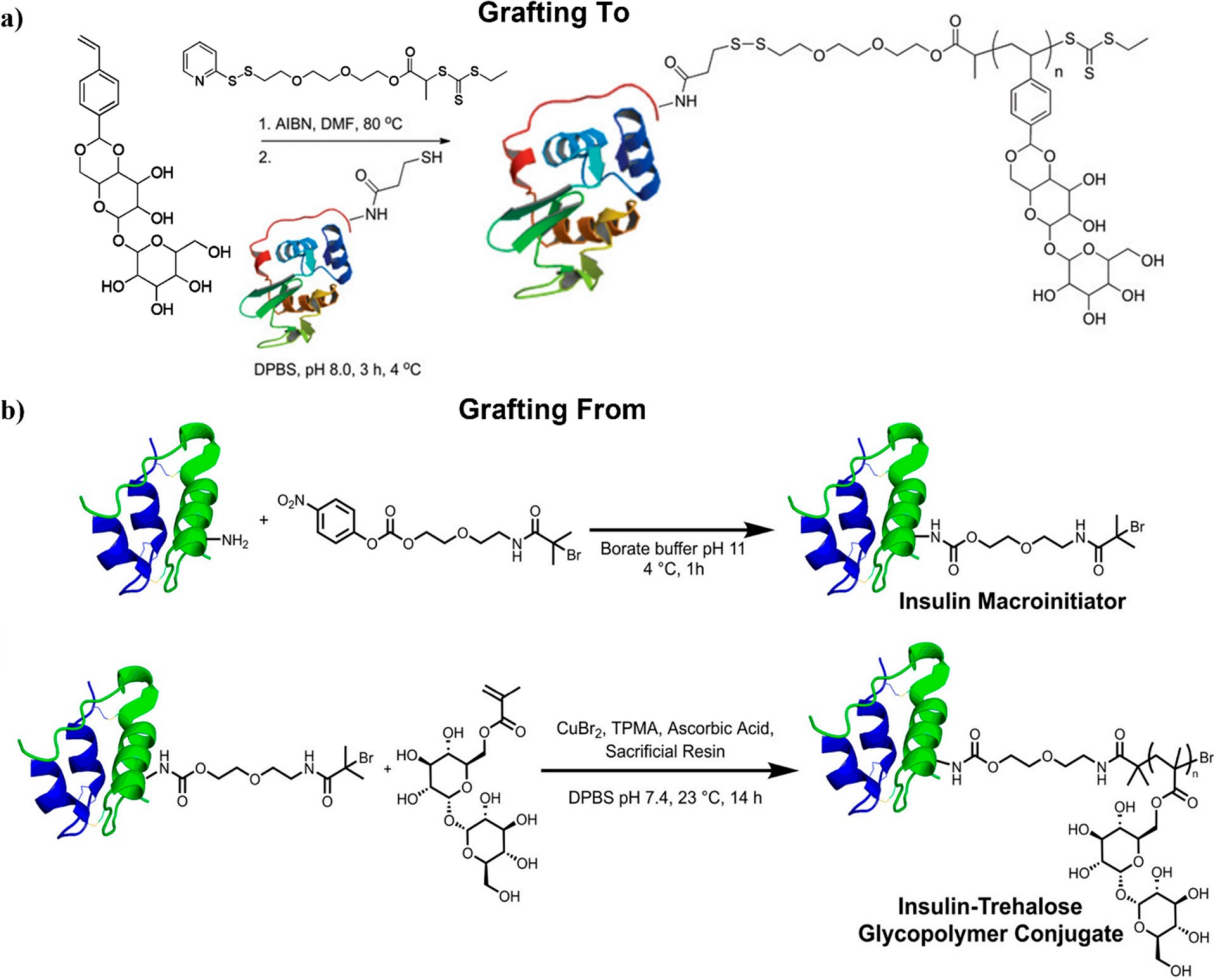
Example of the synthesis of polymer–protein conjugates with trehalose in the side chains using (a) RAFT polymerization of styrenyl acetaltrehalose monomer and conjugation of lysozyme via a “grafting to” approach. Adapted from ref (19). Copyright 2012 American Chemical Society. (b) Insulin macroinitiator synthesis and AGET ATRP of trehalose methacrylate via a “grating from” approach. Adapted from ref (63). Copyright 2018 American Chemical Society.

**Figure 9:**
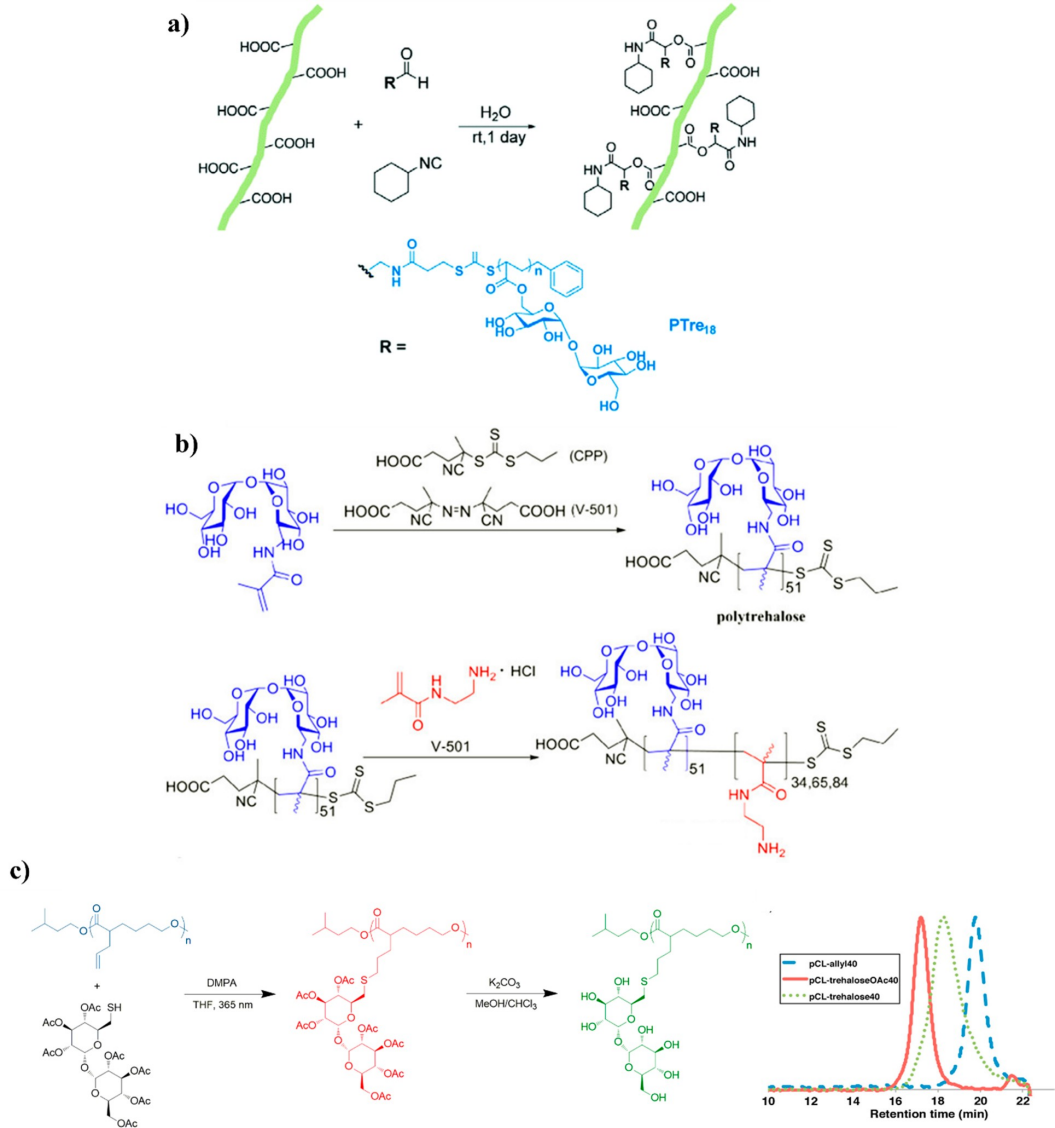
Schematic illustration of (a) surface modification of CNFs with poly(trehalose acrylate) via Passerini reaction. Reproduced from ref (70) with permission from the Royal Society of Chemistry. (b) Synthesis of block copolymers via RAFT polymerization chain extension. Adapted from ref (71). Copyright 2013 American Chemical Society. (c) Synthetic scheme of thiol–ene postmodification of pCL-allyl polymers with acetyl-trehalose and deprotection with GPC characterization of each step. Adapted from ref (20). Copyright 2017 American Chemical Society.

Our group became interested in the field of trehalose polymers, and shortly after, began making numerous contributions to the pendant trehalose design with a particular interest in preparing well-defined protein–polymer conjugates.^19,20,24−26,58−67^ An approach taking advantage of the benefits of reversible-deactivation radical polymerization (RDRP) was the initial focus. A styrenyl monomer bearing a monodiethyl acetal in the para position was reacted selectively in the 4,6 positions by acetalization to afford the styrenyl acetal trehalose monomer in 41% yield. Using a pyridyl disulfide (PDS) functionalized chain transfer agent (CTA), polymers **9** were synthesized via reversible addition–fragmentation chain transfer (RAFT) polymerization.^19^ As is common for controlled polymerization techniques, this method presented many advantages such as low dispersity, possibility to target a specific molecular weight, compatibility with multiple architectures, and high end group retention.^68^ The last advantage was especially important because the PDS group was installed at the ω-chain end for a postpolymerization reaction with proteins to create polymer–protein conjugates (Figure 8a). Inclusion of a short PEG spacer between the PDS and the CTA improved conjugation yields with the protein as visualized by sodium dodecyl sulfate–polyacrylamide gel electrophoresis (SDS-PAGE). Polymerizations proceeded in controlled fashion for 6 h with high conversion, affording a series of polymers with MWs in the 4–50 kDa range and *Đ* values as low as 1.05.^19^ Shortly after, the trehalose monomer and polymer library was expanded to include methacrylate acetal **10**, styrenyl ether **11**, and methacrylate **12**, with each prepared in a few steps with moderate yields. FRP with azobis(isobutyronitrile) (AIBN) at 80 and 65 °C for the styrenyl and methacrylate monomers, respectively, successfully yielded polymers of 10.8–23.4 kDa.^25^ The styrenyl ether monomer synthesis was undertaken without any protection steps, resulting in a mixture of regioisomers that were easily isolated by preparative HPLC.^59^ Four isomers were isolated, with the styrenyl group in position 2, 3, 4, or 6. Regioselectivity could be controlled through a careful choice of base metal counterion for the etherification reaction, with sodium and potassium hydroxide favoring the 4 or 6 position, respectively, and a higher reaction temperature or the use of water as a solvent, raising O6 relative yields. Quantum mechanical calculations confirmed that each isomer maintained the clam shell conformation, important for the stabilizing properties of trehalose.^59^ Stenzel and co-workers also exploited the high chain-end retention of RAFT polymerization to design and synthesize polymer–gold nanoparticle (AuNP)^69^ or polymer-cellulose nanofiber (CNF)^70^ constructs to inhibit microbial adhesion. In the first case, acetylated trehalose acrylate was prepared in three steps with a modest 7% overall yield. Polymerization was conducted at 70 °C in dioxane using a trithiocarbonate CTA, obtaining a polymer with a low MW and dispersity (8.9 kDa, *Đ*: 1.1). Afterward, the polymer was deprotected using sodium methoxide and used to coat AuNPs through thiol–gold interaction.^69^ In the second case, a four step trehalose acrylate synthetic procedure was employed, involving protection and deprotection of hydroxyls with trimethylsilyl chloride. This improved synthetic scheme raised the overall yield to 34%. A CTA bearing an aldehyde in α position was used to afford the trehalose polymer (MW: 10.8 kDa, *Đ*: 1.07), which was grafted to TEMPO-oxidized CNFs via Passerini reaction (Figure 9a).^70^

Among the other advantages of RDRP, the ability to generate well-defined block copolymers has been employed by Reineke and co-workers. A trehalose-methacrylamide monomer was polymerized by RAFT polymerization to form a poly(trehalose) macroCTA from which the chain was then extended with a cationic block to afford polymer **13** and used for gene delivery and stabilization (Figure 9b).^71^ Similarly, diblock terpolymers were readily prepared by polymerizing trehalose-methacrylamide with a variety of comonomers for use in micelle formulations or pH-responsive drug delivery.^72,73^ The ability to generate block copolymers was also exploited by Wang et al. to investigate how the positioning of trehalose monomers within a single-enzyme nanoparticle (SEN) affected protein stabilization. A PEG-macroCTA was used to initiate polymerization of either statistical or block copolymers of *N*-[3-(dimethyl amino)propyl] acrylamide (DMAPA) with acrylamide, trehalose, or sucrose monomers. DMAPA is a cationic monomer that favors protein binding and interaction. To form SENs, the polymers were mixed with the enzyme glucose oxidase (GOx) and then cross-linked by a radical process using trehalose acrylate and bis(acrylamide).^74^

Moving beyond RAFT polymerization, atom transfer radical polymerization (ATRP), and specifically activators generated by electron transfer (AGET) ATRP, has been employed by our group and others to prepare poly(trehalose) polymers. For example, an insulin–poly(trehalose) conjugate was synthesized by installing a nitrophenyl carbonate-activated ATRP initiator at a lysine residue (LysB29), HPLC purifying the singly modified insulin, and using AGET ATRP to “graft from” the protein (Figure 8b).^63^ By growing the poly(trehalose) directly from insulin, both purification and isolation of a singly modified lysine conjugate were streamlined when compared to “grafting to” insulin.^75,76^ AGET ATRP was chosen to polymerize the trehalose monomer because of the mild, aqueous, and room temperature conditions required for the insulin. A sacrificial resin was added so that the polymerization would occur.^63^ Recently, we used AGET ATRP to graft poly(trehalose) to the antibody Herceptin (trastuzumab) and Herceptin antigen-binding fragment (Fab) via a bis-sulfone alkyl bromide initiator, which was chosen as a specific, stable, and irreversible reduction-conjugation handle for disulfide bridging. It was hypothesized that the bis-sulfone might undergo ligand-assisted elimination, giving an alkene that may potentially lead to side reactions and loss of polymerization control. Through careful optimization of polymerization conditions, such as a tris(2-pyridylmethyl)amine (TPMA) ligand equimolar concentration relative to Cu salts or more dilute monomer concentrations, the reaction occurred in a satisfactory controlled fashion, with *Đ* below 1.10. The resulting polymer was then conjugated to Herceptin and Herceptin Fab, and mass spectrometry experiments revealed that, while conjugation of the full antibody led to a heterogeneous mixtures with multiple modification sites, single modifications were achieved for the Fab.^64^ Mantovani and co-workers prepared linear and 4-arm star poly(propargyl methacrylate) polymers via classic ATRP, and azido-trehalose and other sugars were added in a postpolymerization CuAAC reaction.^77^ Morelli et al. used a similar CuAAC postpolymerization modification approach to functionalize azido-bearing poly(disulfide)s with alkyne-trehalose and other sugars. The polymers were prepared by ring-opening disulfide exchange polymerization and underwent the postpolymerization modification with high yield. Both strategies employed post-polymerization modification to allow a direct biological comparison of the various glycopolymers without concern for possible different polymer physicochemical characteristics.^78^

Maynard, Sawamoto, and co-workers also utilized ruthenium-catalyzed living radical polymerization to copolymerize acetylated trehalose methacrylate (AcTrMA) with poly(ethylene glycol) methacrylate (PEGMA) and 1*H*,1*H*,2*H*,2*H*-perfluorooctyl methacrylate (13FOMA) to obtain amphiphilic macromolecules capable of self-assembly in water and organic solvents. The choice of solvent was critical in controlling the polymerization, achieving low dispersity and equimolar monomer incorporation. Initial AcTrMA and 13FOMA polymerizations carried out in toluene yielded polymers with relatively high dispersities (*Đ*: 1.55), whereas switching to 1,2-dichloroethane (DCE) reduced *Đ* to 1.27. However, polymerization time greatly increased, up to 96 h. Ultimately, a 6:4 mixture of toluene/DCE produced lower dispersity (*Đ*: 1.35) polymers in reasonable reaction times. The addition of PEGMA as a comonomer lowered the dispersity even more (*Đ*: 1.26), as the monomer has intermediate polarity that mediated the interaction of the other two comonomers. The polymers were deacetylated using hydrazine hydrate, and self-assembly as monitored by dynamic light scattering (DLS) showed a bimodal distribution of smaller peaks of 10 nm coming from single-chain species and larger peaks at 100–200 nm resulting from interchain assemblies.^61^

Our group has made several efforts to incorporate biodegradable moieties into poly(trehalose) structures. One strategy used RAFT polymerization to copolymerize 5,6-benzo-2-methylene-1,3-dioxepane (BMDO), a cyclic ketene acetal (CKA) which ring opens during polymerization to form degradable esters, with but-3-enyl methacrylate (bMA), an alkene containing monomer. No cross reactivity of the alkene unit was noticed during polymerization, but the final dispersity was relatively high (*Đ*: 1.76), attributed to mismatch reactivity between the monomers. Thiol–ene chemistry was then used to add thiol-trehalose to the alkenes, yielding polymer **14**. The polymer was degradable in basic conditions, with a 59% molar mass loss as shown by gel permeation chromatography (GPC).^60^ Alternatively, biodegradable units were introduced in the polymer chain via ring opening polymerization (ROP) of cyclic esters. Polycaprolactone, polyvalerolactone, polycarbonate, and polylactide with reactive alkene side chains were polymerized with different organocatalyst and cocatalyst systems at room temperature with fast kinetics and low dispersity. Thiol-trehalose was again added during postpolymerization, by thiol–ene chemistry (Figure 9c).^20,62^ The trehalose polycaprolactone **15** underwent hydrolytic cleavage within 24 h in accelerated basic conditions.^20^

Compared to the trehalose backbone strategy, the side chain approach presents some advantages. The ability to use controlled polymerization techniques opens the door for the fine-tuning of MW and dispersity, adding chain-end control and allowing for bioconjugation and the ability to form well-defined random, gradient, or block copolymers along with a larger (co)monomer scope and orthogonality. Moreover, modification of the side chains allows for the introduction of different functionalities as comonomers. Trehalose meth(acrylate) monomers are perhaps the most useful, with them being polymerized by FRP, RAFT polymerization, and ATRP (AGET or Ru-catalyzed) and often not requiring protection steps to achieve controlled polymerization with narrow molecular weight distributions. Nonetheless, monomer yields are still generally very low and polymer backbones are usually not degradable, unless more complex synthetic strategies are employed such as involving the use of CKAs or needing postmodification to introduce trehalose.

### 2.3 Thermoset Resin

**Figure 10:**
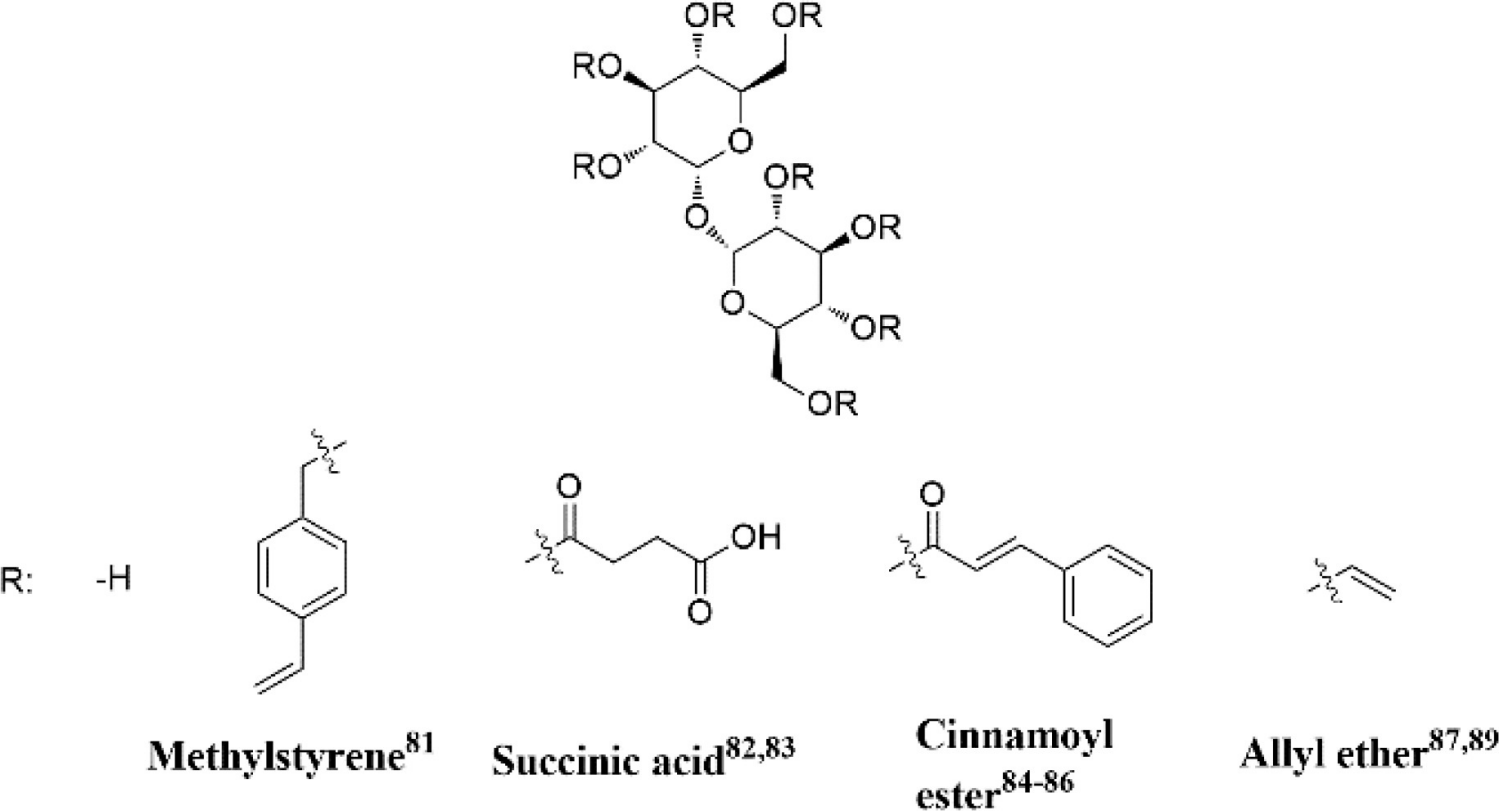
Representative trehalose monomers with different DS for curing and preparation of thermoset resins.

Other than producing linear polymers, trehalose monomers can be cross-linked to form thermoset resins of insoluble polymer networks with outstanding thermomechanical properties (Figure 10). Out of concern for the environment, a focus on producing thermosets from renewable resources has led multiple scientists to replace petroleum-based polymers with biorenewable stocks, such as saccharides.^79,80^

**Figure 11:**
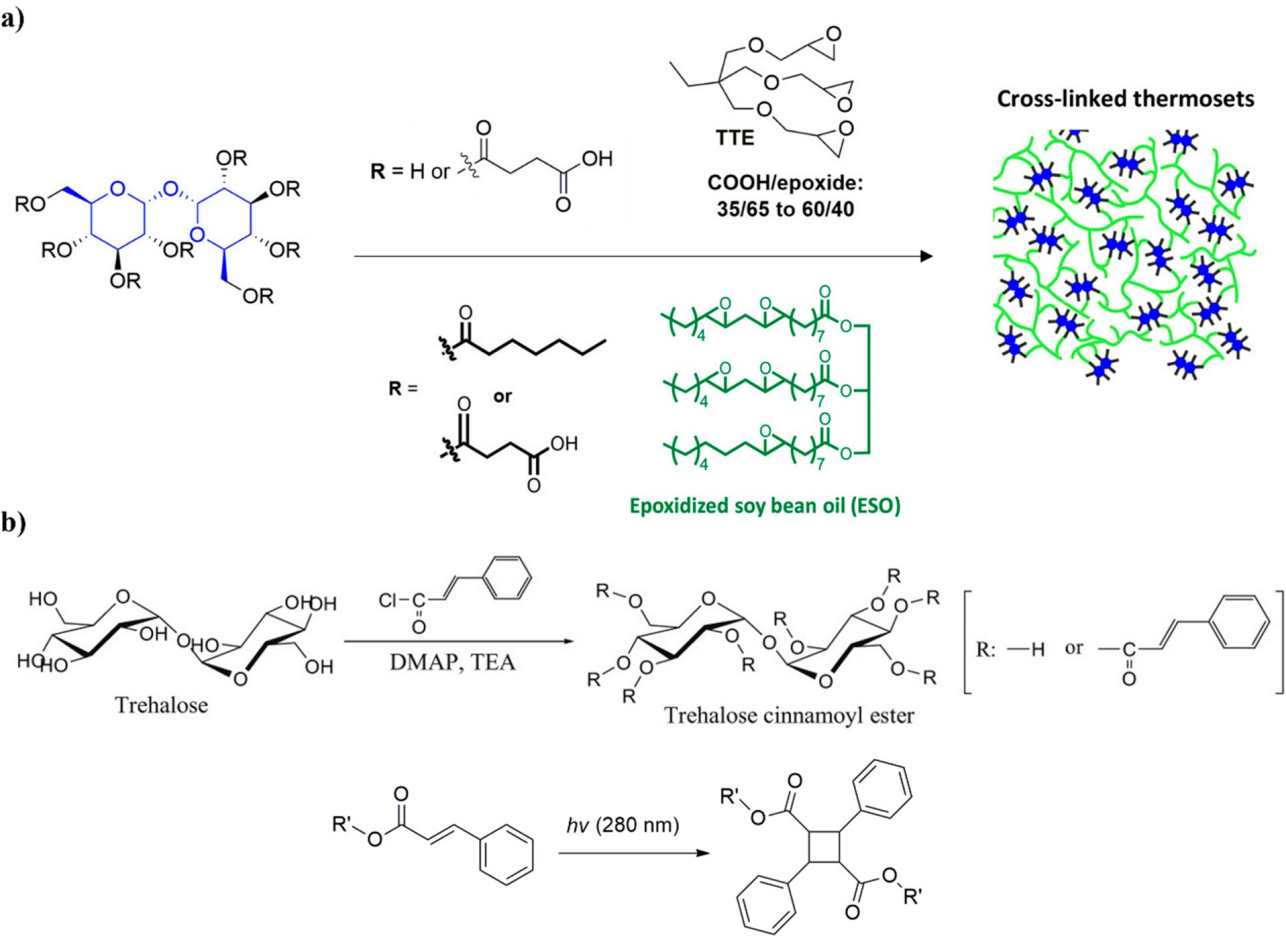
Schematic illustration of (a) trehalose functionalized with succinic anhydride or/and heptanoyl chloride and epoxy resin thermoset synthesis by reaction with TTE or ESO. Adapted from refs (82) and (83). Copyright 2016 and 2018, respectively, American Chemical Society. (b) Synthesis of TCs and photodimerization of cinnamoyl groups. Adapted with permission from ref (84). Copyright 2015 SAGE Publications.

Teramoto and Shibata provided the first example of a thermoset polymer based on trehalose.^81^ Styrenyl moieties were installed on the sugar by reaction with *p*-chloromethylstyrene, with a maximum degree of substitution (DS) of 3.2. The monomer was then cured by applying heat and pressure (200 °C, 29 bar, 30 min), and the thermal properties were analyzed. A correlation between *T*_g_ and DS was found, showing lower *T*_g_ (ranging 118–143 °C) with higher DS. Only 5% of the resin degraded over 50 days, and no further degradation was observed up to 90 days. This was attributed to the hydrolytic stability of the styrenyl backbone.^81^ The Reineke group functionalized trehalose with succinic anhydride for use as a cross-linking hardener for their epoxy-containing trimethylolpropane triglycidyl ether (TTE)-based^82^ or epoxidized soy bean oil (ESO)-based^83^ thermosets (Figure 11a). The properties of the cured thermosets varied greatly, with *T*_g_ values of 63 and 3 °C and tensile strengths up to 47 and 1.3 MPa for the TTE- and ESO-based trehalose thermosets, respectively. In particular, the trehalose/TTE resin showed a high adhesion strength of 3600 psi. The TTE resins were degradable in both basic and acidic conditions, reaching full degradation in a few hours or 1–2 months, respectively, but remained stable at neutral pH. On the other hand, trehalose containing ESO resins were instead stable in both neutral and acidic conditions but quickly degraded in basic media, with higher rates for resins with more unreacted carboxylic groups. The different behavior in acidic media was attributed to the higher hydrophobicity of the ESO moiety compared to TTE, whereas in base the carboxylic groups facilitate water penetration.^83^ The authors noticed that the ESO resins prevented cell adhesion and growth and attributed this to the low elastic modulus, thus proposing the material as a potential antifouling coating material.^83^ Conversely, in a different study, trehalose cinnamoyl ester (TC) smooth thin films promoted fibroblast cell proliferation, with better results than a standard polystyrene culture plate.^84^ TCs were prepared by esterification between trehalose and cinnamoyl chloride, and thin films were prepared by photocuring of the monomer solution, as cinnamoyl undergoes dimerization to form a cyclobutane ring under UV irradiation (Figure 11b). The polymerization is favored with a DS of 4 compared to a DS of 8, due to the larger steric hindrance from the extra cinnamoyl groups in the latter. Photocured TCs showed a *T*_g_ of 91.6 °C.^85^ In a follow up study, unreacted hydroxyl groups of the TCs were further functionalized with 4-(4-hexyloxybenzoyloxy)phenoxy-6-oxohexanoic acid (HBPHA) as a mesogenic unit, yielding a material with a liquid-crystal morphology from 150 to 180 °C. The resulting thin film was found to be biocompatible, and plates coated with the film allowed fibroblast cell attachment and had properties comparable to those of a polystyrene culture plate. Due to the mesogenic characteristics of the material, some of the spindle shaped cells were found to align in a controlled fashion.^86^

An alternative photocuring strategy employs thiol–ene photopolymerization of allyl-etherified trehalose (AT) with various thiols, such as pentaerythritol-based tetrathiol (S4P)^87^ or isocyanurate-based trithiol (S3I).^87,88^ The films were transparent to visible light and presented a *T*_g_ of approximately 27–28 °C in most cases.^87^ Interestingly, the S4P-based film showed a higher tensile strength and modulus but a lower elongation at break than those of the S3I-based film.^87^ To improve the thermomechanical properties, polysilsesquioxanes were included as co-cross-linkers in the production of the S3I-based film, affording organic/inorganic hybrid nanocomposites. The resulting films were transparent and uniform even at the microscopic level. Additionally, the *T*_g__,_, tensile strength, and modulus were higher than those of the organic analogues, increasing with inorganic content.^88^ In a recent report, AT was functionalized with cysteamine hydrochloride to afford aminated trehalose. The monomer was cured with sorbitol polyglycidyl ether (SPE) in the presence or absence of CNFs. The surface of films without CNFs was smooth, and while the addition of CNFs rendered surfaces uneven, they also had the expected effect of increasing the tensile strength and modulus. In the case of trehalose polymers with high amine content and cross-linking, the *T*_g_ was ca. 43.6 °C regardless of the presence of CNFs. At lower amine content and cross-linking, the *T*_g_ decreased from 62 to ca. 50 °C in the presence of CNFs.^89^

### 2.4 Hydrogels/Microgels/Nanogels

**Figure 12:**
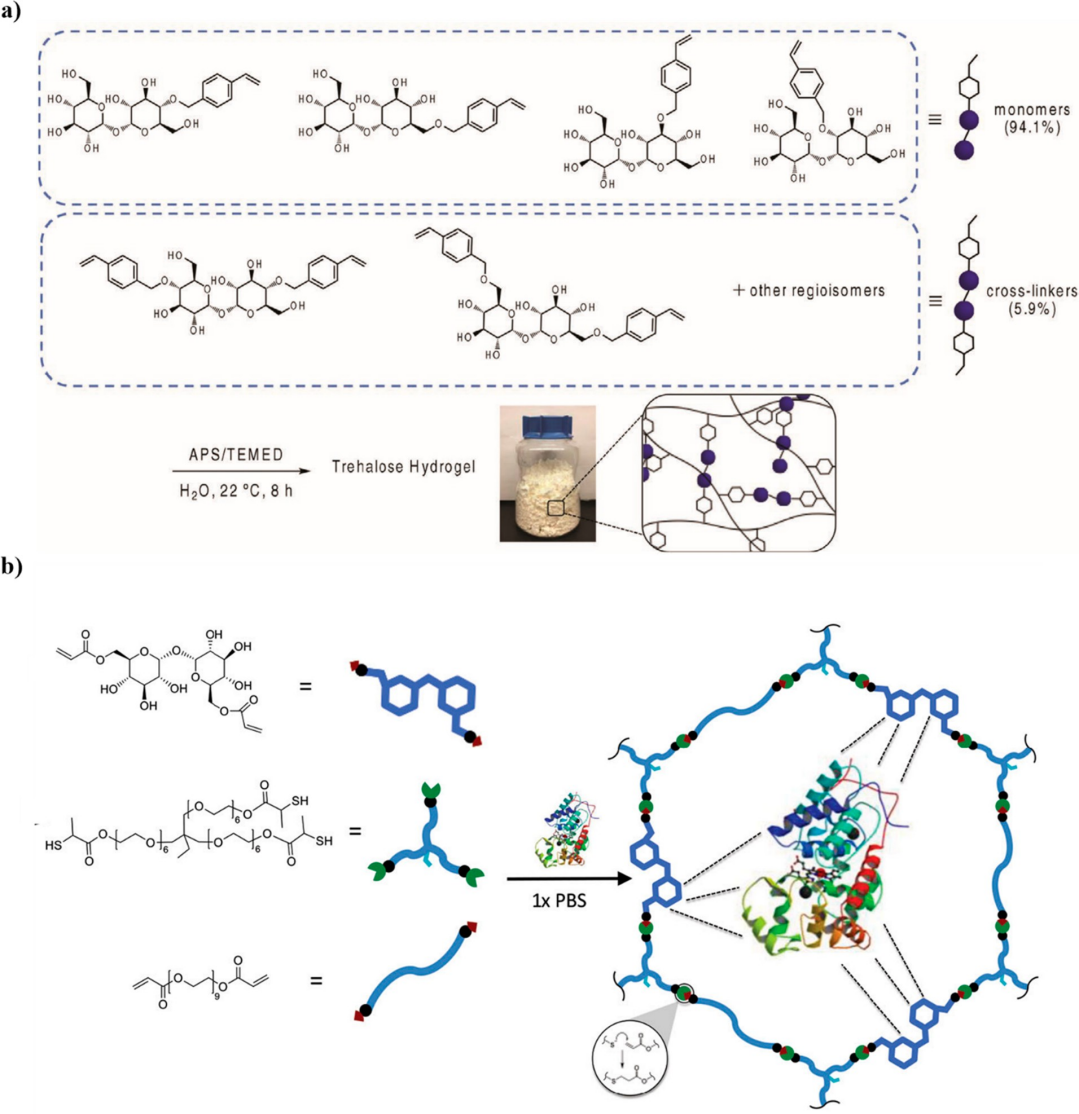
(a) Large scale trehalose hydrogel synthesis using styrenyl ether trehalose monomers and cross-linkers. Reproduced with permission from ref (24). Copyright 2019 Wiley. (b) Trehalose hydrogel for protein delivery prepared by thiol–ene reaction. Reproduced with permission from ref (98). Copyright 2015 Wiley.

Hydrogels are highly cross-linked polymer networks able to trap and retain large amounts of water that have many applications in biomedicine and biotechnology.^90^ Like thermosets, hydrogels containing trehalose have been made. Our group proposed a simple two-step synthesis to prepare trehalose-based hydrogels.^24,65^ Trehalose was modified by etherification with 4-vinylbenzyl chloride, and after precipitation in dichloromethane (DCM) a mixture of mono-, di-, and trisubstituted monomers was obtained. The crude mixture was polymerized directly in water at room temperature using ammonium persulfate (APS) and tetramethylethylenediamine (TEMED) as a co-initiator pair, with the multisubstituted monomer acting as the chemical cross-linker. The purified hydrogel was obtained as a colorless powder, although this first attempt provided only a modest 17% yield.^65^ By increasing the 4-vinylbenzyl chloride to trehalose ratio, greater trehalose modification was achieved, with a preference for the monosubstituted monomer (Figure 12a). The scaled up multigram reaction gave a 76% yield, a large increase from that of the previous synthesis. Many solvent systems were screened to identify a greener alternative to the precipitation step that previously used toxic DCM and hexane. Eventually, ethyl acetate/toluene (2:3) was selected as that afforded the highest yield of 64% after radical gelation, which occurred within 10 min.^24^

The examples discussed above employed chemical cross-linking, but trehalose polymers can also be physically cross-linked to form hydrogels. When well-engineered, these hydrogels can be reversible and even stimuli responsive. For instance, our group synthesized a glucose-responsive trehalose polymer hydrogel for insulin stabilization and delivery, taking advantage of the dynamic covalent bond formed between phenyl boronic acids (PBA) and diol containing molecules. A styrenyl trehalose polymer was prepared by FRP and mixed in phosphate-buffered saline (PBS) with an 8-arm PEG bearing PBA at every end group. A gel formed within 5 min, and it was hypothesized that the multivalency of the trehalose polymer favors gelation since trehalose itself has a very low affinity for PBA. Due to the higher binding affinity of PBA for glucose compared to trehalose, in the presence of glucose, the polymer was displaced, cross-linking was broken, and the hydrogel dissolved in a concentration dependent manner.^66^

**Figure 13:**
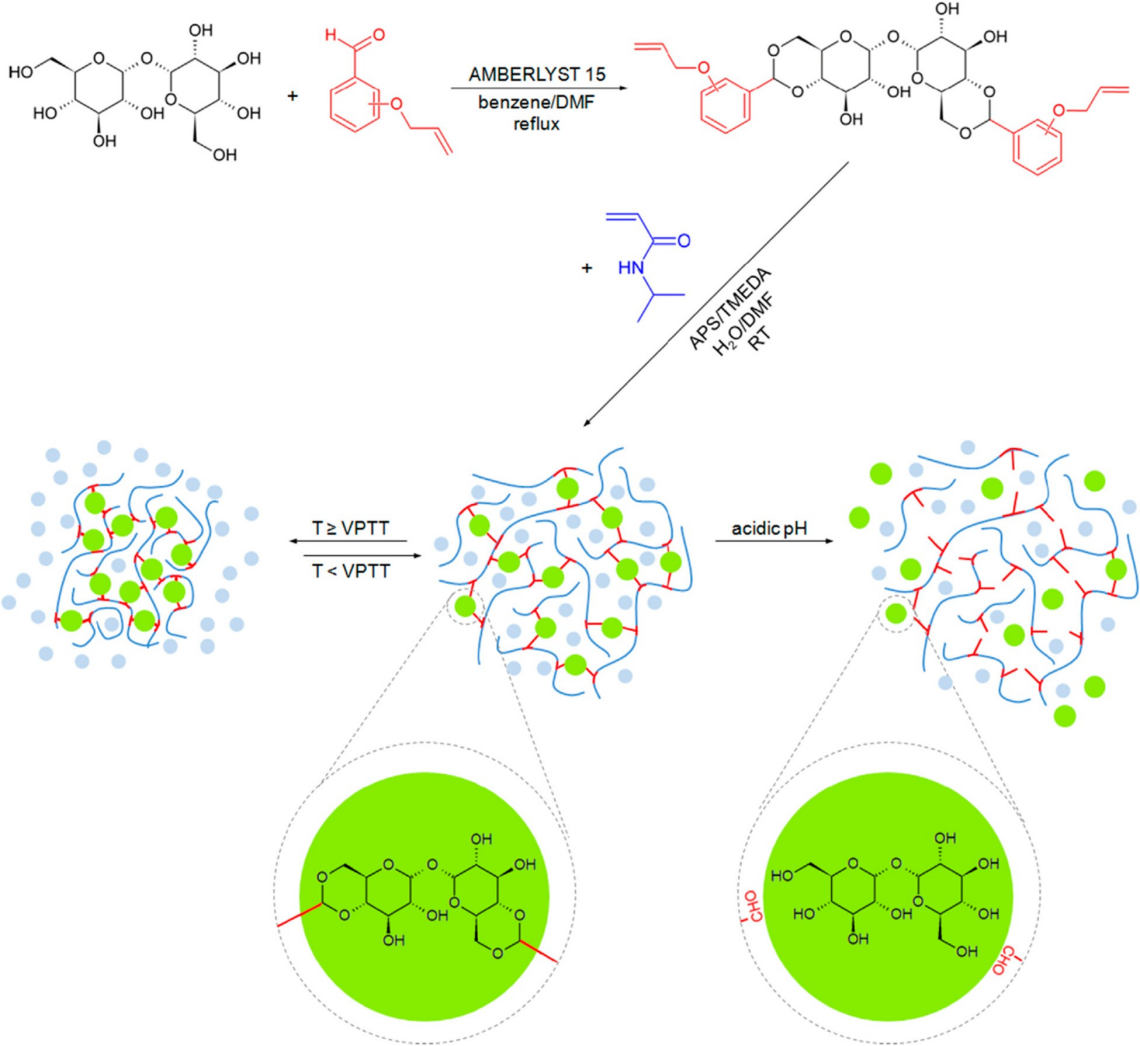
Synthesis of acid-cleavable acetal trehalose hydrogels and their thermoresponsive behavior based on the volume phase transition temperature (VPTT). Reproduced with permission from ref (91). Copyright 2014 Elsevier.

Burek and co-workers designed a series of thermoresponsive and acid degradable hydrogels using modified trehalose as a cross-linker and *N*-isopropylacrylamide (NIPAM) as a monomer.^91−96^ Trehalose was functionalized with 2-, 3-, or 4-allyloxybenzaldehyde to form diacetals regioselectively at the C4 and C6 positions. These trehalose cross-linkers were insoluble in water; thus, water/dimethylformamide (DMF) mixtures were employed for the polymerization, with 1:1 and 2:1 ratios. The TEMED/APS co-initiator pair was used to generate the initial radicals, with TEMED maintaining a basic pH to avoid acetal hydrolysis throughout the 2 h polymerizations at room temperature. The effects of the solvent system, cross-linker identity, and mole percent on the lower critical solution temperature (LCST) and volume phase transition temperature (VPTT) of the hydrogels were studied. Due to the low mole percent of trehalose cross-linker, VPTTs were similar to those of NIPAM homopolymer hydrogels with a range of 31.5–34.5 °C until the mole percent was increased to 4%, when the VPTT unexpectedly decreased to 29 °C. The authors hypothesized that water preferentially interacts with trehalose moieties, resulting in weakened hydrogen bonds with the NIPAM amide groups. The same characteristics also influenced swelling abilities, with low cross-linking, 2-isomers, and higher water content in the solvent system leading to higher swelling capacity. Due to the presence of acetals in the cross-linker, the hydrogel degraded within hours in an acidic solution at room temperature, although no degradation occurred at acidic pH above the VPTT, due to the shrinkage of the hydrogel and masking of the acetals (Figure 13).^91^

To obtain a hydrogel able to degrade at physiological temperatures, hydrophilic comonomers, such as acrylamide (AAm), *N*-(2-hydroxyethyl)acrylamide (HEAAm), and *N*,*N*-dimethylacrylamide (DMAAm), were added in the polymerization feed. Using 13–25 mol % of these comonomers, hydrogels with VPTTs of 37–42 °C were obtained, with HEAAm and AAm containing hydrogels showing the highest VPTT values. With increased hydrophilic comonomer content, swelling capacities and degradation rates also increased, making degradation possible at physiological temperature.^92^ Among other parameters that were altered to tune and modify thermomechanical and degradations properties, a different trehalose comonomer, 4,6-*O*-acrylidene-α,α-d-trehalose, was prepared and found to be water-soluble, eliminating the need for DMF during the polymerization. Moreover, it enabled a higher overall trehalose content for protein stabilization, although incorporation was much lower than the theoretical feed content (up to a 75% difference).^95^

Alternatively, hydrogels with an even higher trehalose content, up to 51.7 wt %, were prepared to treat neurodegenerative diseases, with trehalose as the drug being delivered.^96^ To ensure these hydrogels would also be degradable at basic pH, an ester moiety was added to the trehalose cross-linker.^93,96^ The degradation characteristics could additionally be controlled by the nature of the linker in the para and meta positions of the acetals and by the hydrophilicity of comonomers used.^93,94^ A final set of degradable chitosan hydrogels was prepared using a diiodo-trehalose derivative as the chemical cross-linker; these hydrogels could be fully biodegraded in 96 h by trehalase.^97^

Interestingly, O’Shea et al. developed tricomposite hydrogels by thiol–ene reaction using enzyme derived diacrylate trehalose, PEG diacrylate, and trimethylolpropane ethoxylate thiolactate as a thiol-bearing cross-linker (Figure 12b). Within a few minutes of mixing, hydrogels with varied trehalose contents were prepared and their rate of degradation increased proportionally with trehalose amount. Using attenuated total reflection Fourier transform infrared spectroscopy (ATR-FTIR), they found that the signal strength of the hydroxyl hydrogen bond was linearly dependent on trehalose content. Moreover, hydrogels in the semidry state were found to possess more robust mechanical properties, such as stiffness and tensile strength, compared to fully hydrated gels, and complete dehydration led to materials with properties comparable to analogue gels not containing trehalose, confirming the importance of the carbohydrate in hydrogen bond formation and organization.^98^

Related to hydrogels in composition and applications, nanogels and microgels are defined as highly cross-linked hydrophilic polymers that form particles at the nanometer or micrometer scale, respectively. Our group synthesized trehalose-based nanogels for the stabilization and delivery of glucagon, an unstable peptide hormone used in the treatment of hypoglycemia.^58^ Briefly, a PDS containing trehalose copolymer, poly(pyridyl disulfide methacrylate-*co*-trehalose methacrylate) (PDSMA-*co*-TrMA), was prepared by FRP of the respective monomers. Cross-linking with a 1 kDa PEG-dithiol yielded nanogels of about 9 nm regardless of the amount of cross-linker, although the size could be controlled by tuning the polymer concentration. By installing two thiols on glucagon using dimethyl-3,3′-dithio-bis(propionimidate), the peptide itself could be used as a cross-linker to form nanogels in less than 2 h, with a 60–70% yield. A higher pyridyl disulfide methacrylate (PDSMA) content, a polymer concentration of 0.5−1 mg mL^–1^, and a 5:1 thiol ratio of polymer to glucagon resulted in more uniform particles.^58^ Another example was provided by Wandzik and co-workers, where an acrylidene trehalose monomer and its diacrylidene version, as a cross-linker, were copolymerized with NIPAM to form thermoresponsive microgels by surfactant-free precipitation copolymerization. The microgels had diameters in the 200–400 nm range, dispersities < 0.1, and shrinking abilities above their VPTT (ca. 29 °C). However, when dispersed in solutions with ionic strengths of 0.165 M, such as in Dulbecco’s modified Eagle medium (DMEM) cell culture media, the microgels aggregated into a macroscopic hydrogel above their VPTT.^99^

## 3 Applications of Trehalose Materials

### 3.1 Protein and Peptide Stabilization and Delivery

**Figure 14:**
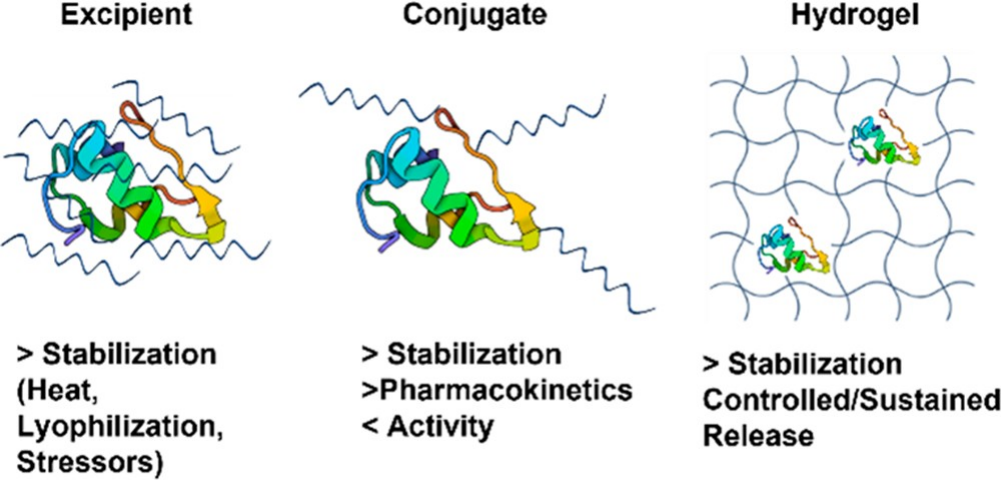
Properties of trehalose polymers as excipients, conjugates, or hydrogels for protein stabilization.

Based on the known stabilization ability, hydrophilicity, and biocompatibility of trehalose as a small molecule, many groups hypothesized that incorporating trehalose into a polymer would aid in drug solubility and prevent the aggregation, denaturation, and degradation of proteins. In the following section, the ability of trehalose polymers to stabilize proteins and peptides as excipients, conjugates, and hydrogels (Figure 14) will be discussed.

#### 3.1.1 Excipients

Linear or star homopolymers and copolymers of trehalose have been studied as excipients for different protein drugs, although all have utilized pendant trehalose monomers rather than incorporating trehalose into the backbone.

**Figure 15:**
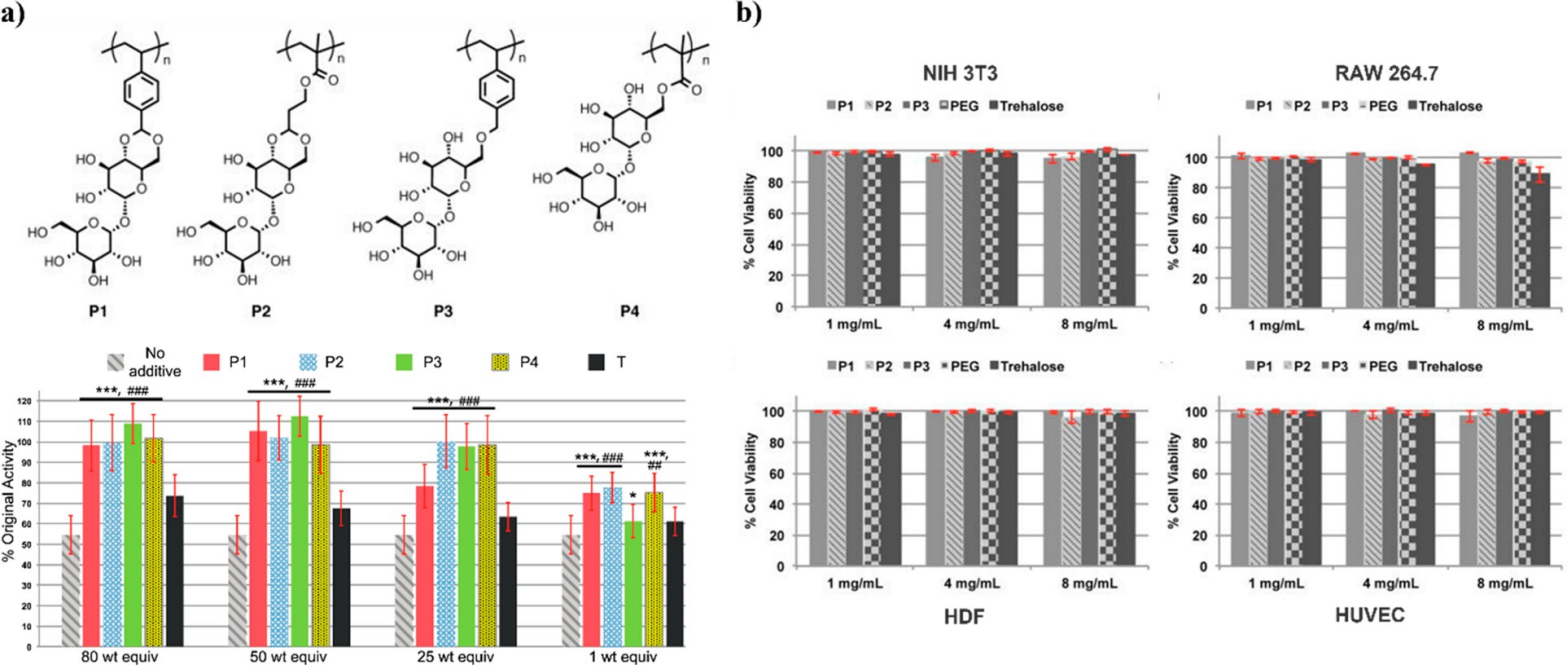
(a) Trehalose polymers and activity of stabilized HRP incubated at 70 °C for 30 min. (b) Cytotoxicity assay of P1–P3 and 20 kDa PEG with cell lines: NIH 3T3, RAW 264.7, HDF, and HUVEC. Reproduced from ref (25). Copyright 2013 American Chemical Society.

The earliest published use of trehalose polymers specifically to stabilize proteins as an excipient and as a protein polymer conjugate came from our group in 2012.^19^ RAFT polymerization of styrenyl trehalose yielded polymers that stabilize hen egg white lysozyme (HEWL) against heat (90 °C for 1 h, 100 mol equiv of polymer) and lyophilization (10 cycles, 1 or 100 mol equiv of polymer). The HEWL activity after exposure to these stressors was vastly improved with added trehalose polymer, up to 100% activity as compared to less than 20% activity without polymer added. Furthermore, the polymeric form was considerably better than trehalose at stabilizing the enzyme, although comparable stabilization ability was found for the excipient or conjugate form.^19^ This study was rapidly expanded to include other trehalose side chain polymers.^25^ The derived polymers, as well as small molecule trehalose, were applied in 1–80 weight equivalents (wt equiv) to horseradish peroxidase (HRP), β-galactosidase (β-gal), and GOx. The percent original activity of β-gal after three lyophilization cycles or of HRP (Figure 15a) and GOx after heating (70 °C for 30 min) clearly showed that all of the polymer excipient formulations, except for 1 wt equiv of pTrMA with β-gal, significantly increased the remaining enzyme activity (60–100% HRP, 50–100% β-gal, and 80–95% GOx activity) relative to no excipient or trehalose. Moreover, the polymers were noncytotoxic in vitro on four different cell lines up to 8 mg/mL (Figure 15b)^25^ and were later found to also be nontoxic in vivo.^67^

**Figure 16:**
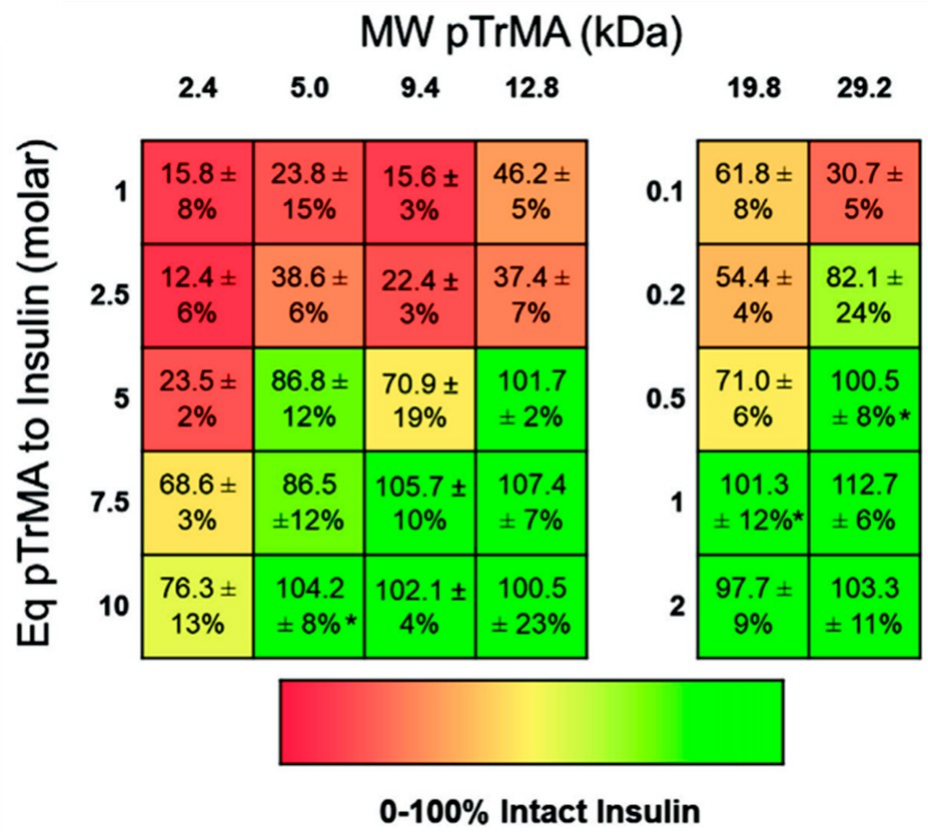
Percentage of intact insulin stabilized with poly(trehalose methacrylate) (pTrMA) with different MWs and concentrations incubated at 37 °C for 3 h. Reproduced with permission from ref (26). Copyright 2021 Wiley.

Counterintuitively, initial experiments with trehalose polymers did not appear to have improved stabilization with increasing MW^19^ even though concentration clearly played a critical role in stabilizing proteins.^19,25,59^ Eventually, the stabilization effect was confirmed to be MW dependent for trehalose poly(ester)s.^20^ Pelegri-O’Day et al. found that, while keeping the total amount of polymer or trehalose in solution constant, increasing the MW of trehalose-based polymer excipients resulted in more stable protein formulations.^20^ A later study with pTMA showed that the molecular weight effect was only observed at lower concentrations of polymer, with larger polymers requiring a significantly lower concentration in order to fully stabilize the protein insulin when compared with smaller polymers (Figure 16).^26^ Due to the polymer backbone connecting individual trehalose molecules, the likelihood of a higher concentration of trehalose molecules in the polymer interacting with the protein surface is increased. This effect is named multivalency. Taking the effect of both MW and concentration into account, trehalose polymer formulations were optimized to reduce the total amount of polymer needed to stabilize proteins.^26^

After exploring the initial range of model enzymes, researchers began employing trehalose polymers to improve the formulation properties and stability of therapeutically relevant drugs: insulin,^26,59,62,63^ granulocyte colony stimulating factor (GCSF),^20,60^ and probucal^73^ as well as antibodies.^77^ Trehalose polystyrene,^59^ polymethacrylate,^26,63^ polycaprolactone, polyvalerolactone, polycarbonate, and polylactide polymers^62^ were all able to maintain fully intact insulin (97–100%) despite heat and agitation (37 °C and 250 rpm for 3–4 h). Although these polymers have yet to be compared in a single experiment, Pelegri-O’Day et al. found that, with 10 wt equiv of the ROP degradable polymers, there was no difference in insulin stabilization, indicating that the side chain trehalose was more important to the stabilizing properties than the polymer backbone was. Additionally, Messina et al. found that all of the different regioisomers of poly(trehalose styrenyl ether) fully stabilized insulin to mechanical agitation.^59^ Thus, the backbone and regioisomer may not be as critical factors as the polymer molecular weight and concentration.

An additional therapeutic that has been studied with a range of different trehalose polymers is GCSF, a particularly unstable protein. The degradable poly(trehalose caprolactone) maintained GCSF activity even after both mild (4 °C for 1.5 h) and aggressive (60 °C for 30 min) temperature changes with 100 wt equiv as measured by improved proliferation of murine myeloid leukemia NFS-60 cells, 168% and 179%, respectively, which hold comparison with fresh GCSF, showing 150–180% proliferation.^20^ In a similar heating assay (40 °C for 30 min), a RAFT copolymerized BMDO-trehalose copolymer maintained GCSF activity at 66% with 10 wt equiv and 51% with 500 wt equiv.^60^ While this was better than no excipient (ca. 30% activity), the degradable copolymer results were inferior to the nondegradable poly(trehalose styrenyl acetal) which resulted in 75–100% remaining GCSF activity depending on the weight equivalents used.^60^ While all three polymers were found to be noncytotoxic up to 1 mg/mL (primary human umbilical vein endothelial cells (HUVECs)^20^ or human dermal fibroblasts (HDFs) and murine myeloblasts NFS-60),^60^ the degradation products of the BMDO-trehalose copolymer reduced cell viability to 74%. Similarly to how the BMDO-trehalose copolymer better stabilized GCSF at lower weight equivalents than higher ones, Mantovani’s group found that linear and four-arm methacrylate-based trehalose polymers were better at stabilizing a highly concentrated model monoclonal antibody (mAb1, 50 mg/mL) against heat (25 and 40 °C for 7 weeks) with a lower mole equivalent of polymer (1 and 100 vs 200 and 300).^77^ The reason for this is uncertain, but it demonstrates that various weight equivalents should be studied. Indeed, our group explored the effect of polymer concentration and MW of poly(trehalose methacrylate) on the stabilization of intact insulin, determined by HPLC analysis. This study showed that, for insulin, increasing the MW or concentration led to greater insulin stability against environmental stresses (Figure 16).^26^ Based on the similarities in stabilization properties across polymer backbones, it seems likely that excipient formulations of the other trehalose polymers could be similarly optimized to reduce the amount of polymer in solution.

#### 3.1.2 Conjugates

Another application of trehalose polymers is in protein–polymer conjugates. Conjugating the polymer directly to the protein could further improve the stabilization of the protein. The polymer may extend the half-life of the biomolecules in vivo as an additional advantage: conjugation of PEG, or PEGylation, has been extensively shown to lengthen the half-life of proteins, including some already on the market such as Somavert, PEGasys, and Neulasta.^100,101^ Beyond PEG, poly(oxazoline), poly(*N*-(2-hydroxypropyl)methacrylamide), and other polymers have also been used to improve the pharmacokinetics (PK) of biologics, so trehalose polymers were anticipated to improve PK.^102^

Thus far, the only reports of protein conjugates made with trehalose polymers have come from the Maynard lab, using diverse conjugation methods, proteins, and trehalose polymers. The first publication focused on modification of HEWL.^19^ “Grafting to” with RAFT synthesized styrenyl trehalose polymer (MW: 8.0–49.5 kDa) formed the conjugates. Conjugation improved the enzyme stability against both heat (1 h at 90 °C) and lyophilization (10 cycles) with up to 100% or 81% HEWL activity, respectively, relative to 17% and 18% of protein alone. The conjugates were more active after lyophilization than polymer as excipient or trehalose, while the conjugate and excipient were comparable after heat stress.

**Figure 17:**
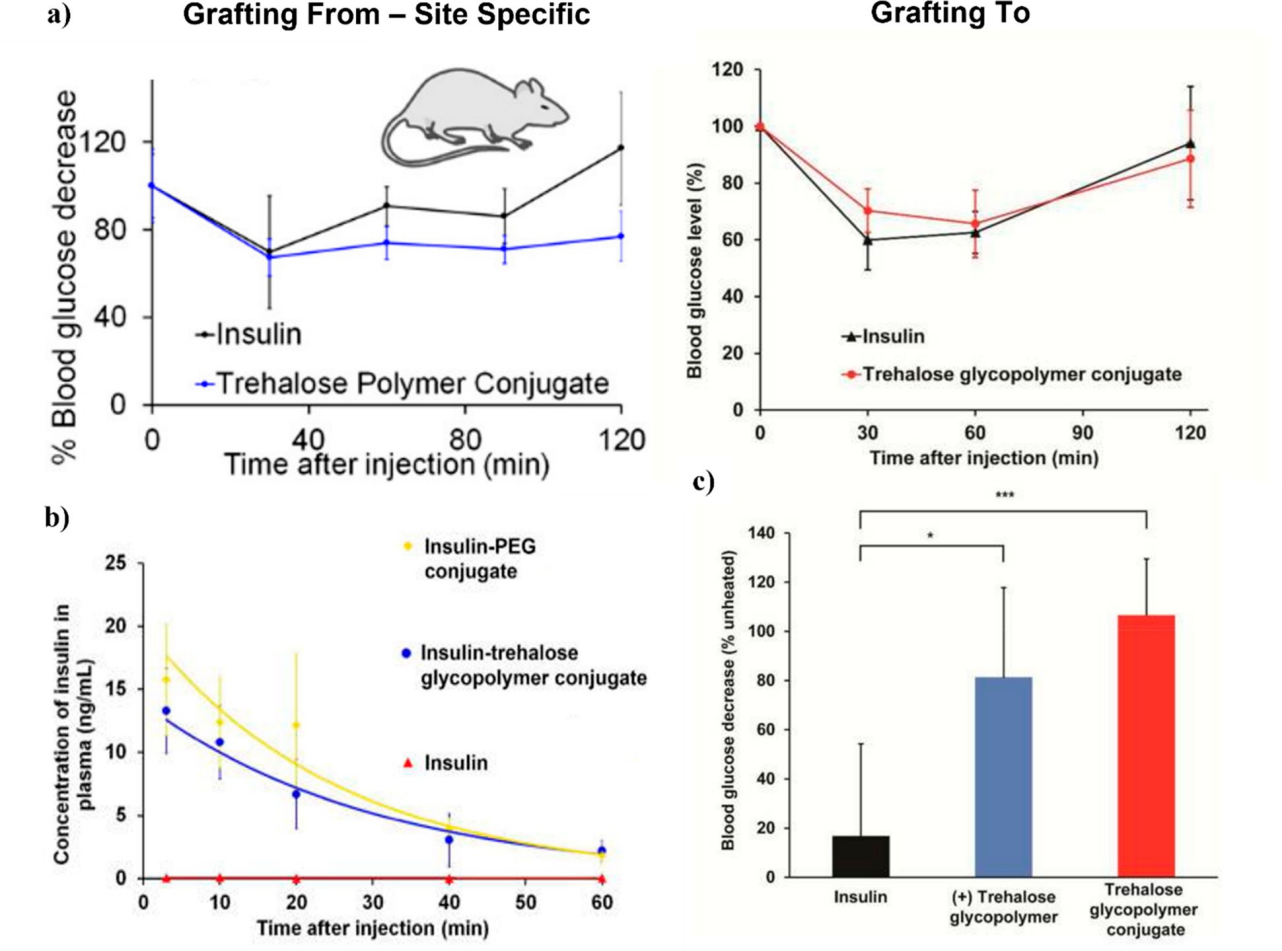
(a) Blood glucose levels in fasted mice after i.v. injection with unmodified insulin, grafting from and grafting to an insulin trehalose polymer conjugate. (b) Pharmacokinetics study of insulin and polymer conjugates. (c) Activity of heated insulin, insulin with trehalose glycopolymer excipient (2 mol equiv), and insulin-trehalose polymer conjugate (90 °C, 30 min) relative to unheated samples during ITT in mice. Reproduced from refs (63) and (67). Copyright 2018 and 2017, respectively, American Chemical Society.

This work was expanded to focus on one of the most widely used therapeutic proteins, insulin, both as a nonspecific “grafted to” conjugate^67^ and as a site-specific “grafted from” conjugate.^63^ The conjugation approaches relied on reductive amination or nucleophilic substitution, respectively. In the second case, the greater nucleophilicity of lysine B29 over the N-terminal amines was exploited by increasing the reaction pH from 8.0 to 9.5 in order to favor single modification of insulin using a nitrophenyl carbonate-activated ATRP initiator as described above (Figure 8b). While the dose of insulin required for each conjugate was higher than that of native insulin, the site-specifically modified insulin required only a 3-fold dosage as compared to the 5-fold dosage of the original conjugate (16 vs 48 vs 80 μg/kg) in order to lower glucose concentrations in mice comparably (Figure 17a). Excitingly, both insulin conjugates stabilized insulin against an accelerated heat stress (90 °C for 30 min) better than unmodified insulin did; by insulin tolerance tests (ITT) in mice, the conjugate retained 100% activity after heat treatment in vivo, while the unmodified protein had 17% activity (Figure 17c).^67^ The longer insulin plasma lifetime of the conjugate compared to insulin was confirmed in mice. The trehalose polymer prolonged the plasma lifetime in a comparable fashion to a similarly sized PEG conjugate, suggesting that the polymers have that advantage of PEG (Figure 17b).^67^

Disulfide bonds were exploited for nonspecifically or site-selectively conjugated trehalose polymers onto an antibody and Fab, respectively.^64^ The conjugation of multiple 16 kDa trehalose polymers to Herceptin and the single 23 kDa trehalose polymer to Herceptin Fab did decrease binding affinity as indicated by ELISA, likely due to polymer steric hindrance: conjugates had higher EC_50_’s relative to the unmodified antibody and Fab, 0.90 vs 0.26 nM and 2.74 vs 0.56 nM, respectively. However, conjugation of the trehalose polymer significantly increased the stability of both the antibody and Fab against heat stress (75 °C for 1 h) with around 50% soluble antibody or Fab conjugate rather than 0% soluble unmodified.

All of the above protein–polymer conjugates provided the greater protein stability that is expected from trehalose polymers. Additionally, comparing the stability of conjugates to trehalose polymer and trehalose small molecule excipient formulations has shown some improvements in stabilization.^19,63^ For insulin, polymer conjugation also enhanced the circulation time of the biomolecule and prolonged the effect of treatment.^63,67^ Similar results are anticipated for other proteins. As expected, decreased bioactivity was the main drawback observed from conjugation,^63,64,67^ but site-selectivity provided some improvement. Similar to PEG conjugates, improved protein half-life is expected to help mitigate the loss of activity, achieving a balance between the two properties. Additionally, it is likely that a more conscious selection of conjugation sites could further reduce the loss in bioactivity, as thus far conjugation sites have been chosen for their accessibility as site-selective points of modification without considering the effects on the biomolecule activity. Alternatively, the use of self-immolative linkers to form polymer/protein conjugates, that release the native protein following certain stimuli, could also mitigate the loss of activity.^103,104^

Interestingly, polymers with trehalose in the backbone have never been tested as an excipient or conjugate for protein stabilization but only for delivery of genetic material as discussed below. In the conjugate case, this might be due to a lack of general conjugation strategies that can be applied to a variety of backbone polymers because of the difficulty in obtaining mono-end-functionalized (i.e., semitelechelic) polymers. A study with a direct comparison of the two strategies could help elucidate the structure–activity relationship of the polymer and inform future advancements.

#### 3.1.3 Hydrogels

Hydrogels should allow both the immobilization of proteins as well as controlled or sustained release through passive diffusion or through network degradation and dissolution. They present some common advantages of drug delivery systems compared to simple linear polymers, such as ease of functionalization, responsiveness to stimuli, degradability, and the possibility to deliver multiple drugs at the same time. Additionally, it can be expected that incorporating trehalose into hydrogels would provide the same or better protein stabilization observed with linear trehalose polymers. Thus, hydrogels have been explored for protein stabilization.

**Figure 18:**
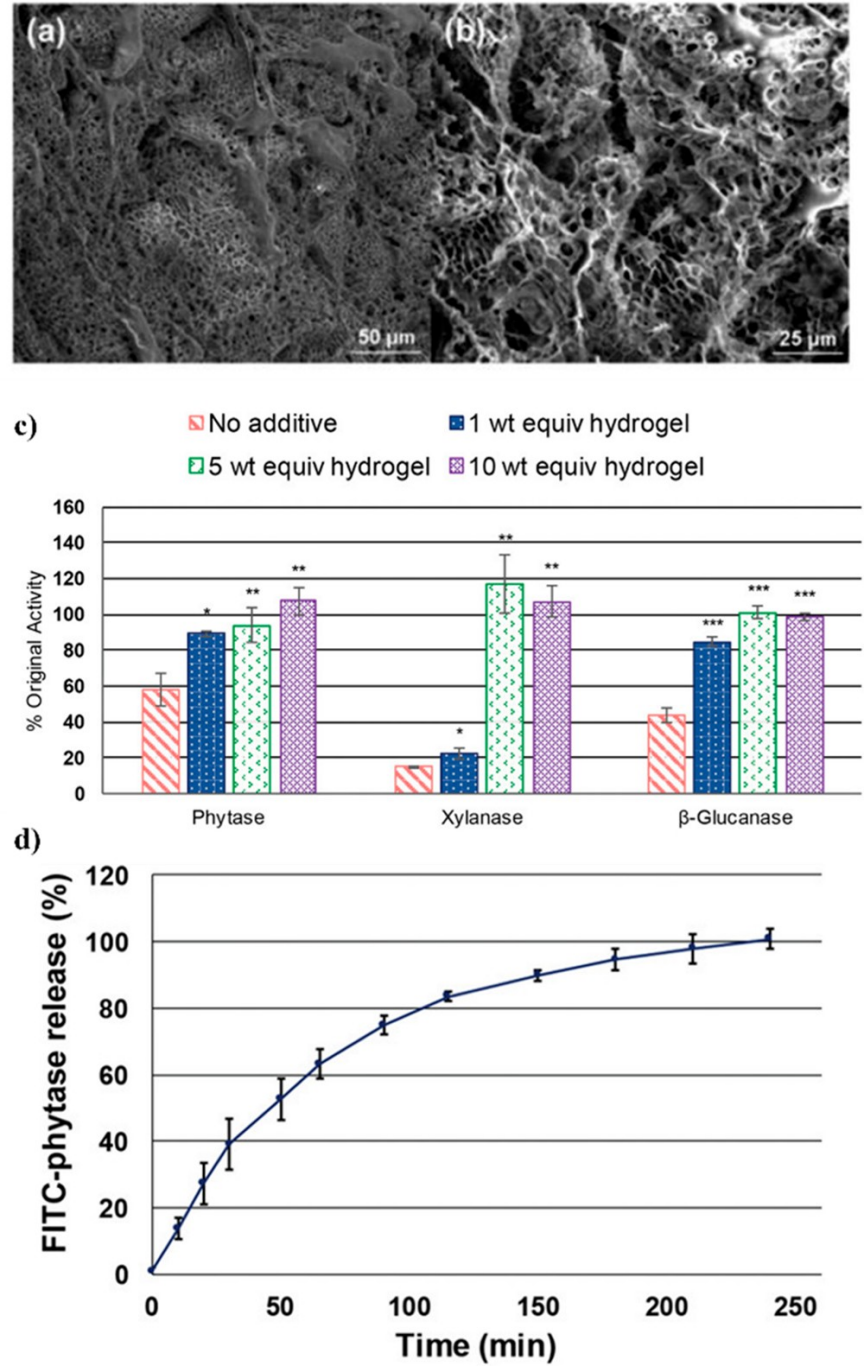
SEM images of trehalose hydrogel at (a) 500× magnification and (b) 1000× magnification. Reproduced from ref (65) with permission from the Royal Society of Chemistry. (c) Activity of phytase, xylanase, and β-glucanase loaded in trehalose hydrogels at various concentrations after incubation for 1 min at 90 °C. (d) Percent cumulative release of loaded fluorescein isothiocyanate (FITC)-labeled phytase from trehalose hydrogels. Reproduced with permission from ref (24). Copyright 2019 Wiley.

Our group and the Langer group simultaneously published two different routes for creating enzyme-stabilizing trehalose hydrogels. Our system used styrenyl or multistyrenyl trehalose as monomer and cross-linker, respectively, and focused more on the protein stabilization.^65^ Langer’s group utilized trehalose diacrylate as part of a three-component system with diacrylate-PEG and trimethylolpropane ethoxylate thiolactate (TMPE-TL) and explored protein release kinetics.^98^ Our group utilized a styrenyl trehalose hydrogel to stabilize phytase, which is an enzyme important to agriculture feed stocks. Variable pressure scanning election microscopy (SEM) was used to characterize the hydrogel, and the images revealed micrometer-sized pores which could easily fit the enzyme (Figure 18a,b). Thus, phytase was entrapped within the network structure at 1, 10, and 40 wt equiv of hydrogel to protein. The trehalose hydrogel was able to protect phytase during exposure to feedstock production-relevant conditions (90 °C, 1 min, 53 wt % water), maintaining 80–100% activity as compared to 39% activity for phytase alone. The best performing formulation (10 wt equiv) was used to study release kinetics, and ca. 80% of the phytase was released in 6 h from the hydrogels by passive diffusion with no agitation.^65^ An expansion of this work tested additional feedstock relevant enzymes (phytase, β-glucanase, and xylanase).^24^ As with the original work, the enzymes were encapsulated in the trehalose hydrogel, exposed to 90 °C for 1 min with 50 wt % water, and then tested for activity. Similar to the original work, with 10 wt equiv, >98% activity was maintained with all enzymes, while only 15–58% enzyme activity was observed when the protein was tested alone (Figure 18c). Notably, phytase and xylanase activity was increased to above 100% in the presence of the gel, possibly due to the gel network and/or trehalose scaffold stabilization enhancing substrate binding or stabilizing the proteins in the activity assay conditions. These results were also compared to the stability of the enzymes in the presence of the same amount of molecular trehalose (0.54, 2.7, and 5.4 wt equiv), and only the highest concentration consistently retained any significant amount of activity (65–100%) relative to the enzymes alone; all hydrogel concentrations outperformed the equivalent concentrations of free trehalose. Importantly, similar to the original work, sustained quantitative release of phytase was achieved within 4 h at 37 °C (Figure 18d), which is a relevant time frame for the average feed transit in the small intestine of pigs.^24^

**Figure 19:**
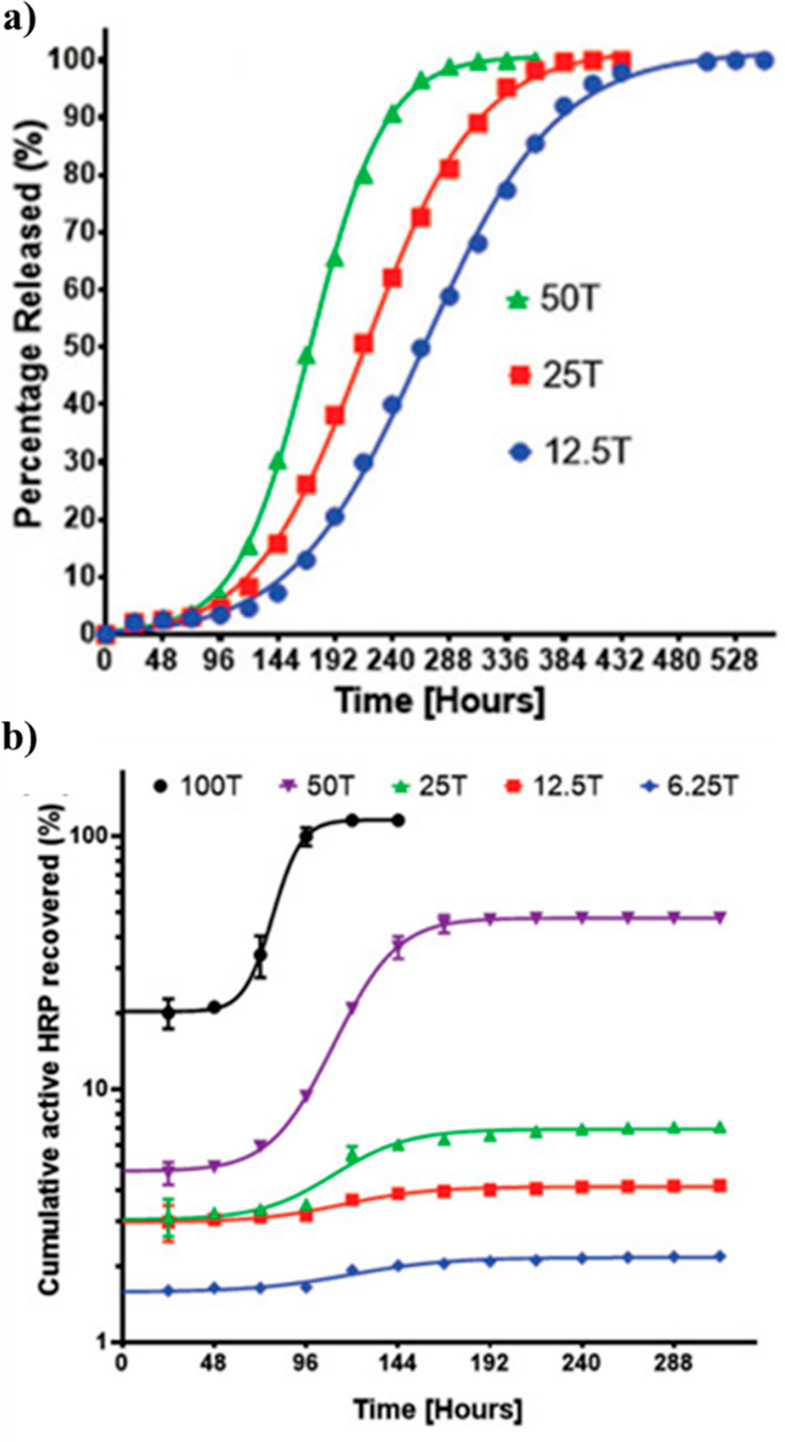
(a) Cumulative FITC-ovalbumin release profiles from various percentage compositions of trehalose within hydrogels. (b) Cumulative HRP recovery for hydrogels with various percentage compositions of trehalose. Time axis represent the amount of hours the loaded hydrogel was heated at 37 °C before HRP was recovered. Reproduced with permission from ref (98). Copyright 2015 Wiley.

Langer and co-workers developed three-component trehalose/PEG/TMPE hydrogels with varying amounts (6.25–100% diacrylate component) of trehalose incorporation, which were found to have faster protein release with increasing trehalose content for both ovalbumin (OVA) and IgG proteins, despite the large difference in size (Figure 19a).^98^ This, along with a triphasic release profile, suggested that the initial diffusion release gives way to network degradation-based release and that the higher the amount of trehalose component, the faster this degradation occurs. Furthermore, HRP was encapsulated in the hydrogel, then exposed to heat (37 °C for up to 12 days), and subsequently recovered to test activity and showed that a higher trehalose content results in higher recovered activity (100% activity for gel with maximum trehalose content vs 50% for gel with half the amount of trehalose vs 7% for gel with 25% amount of trehalose) (Figure 19b). Conversely, the hydrogels with less trehalose content could destabilize the protein, because hydrolysis of the network exposed carboxylate groups.^98^

**Figure 20:**
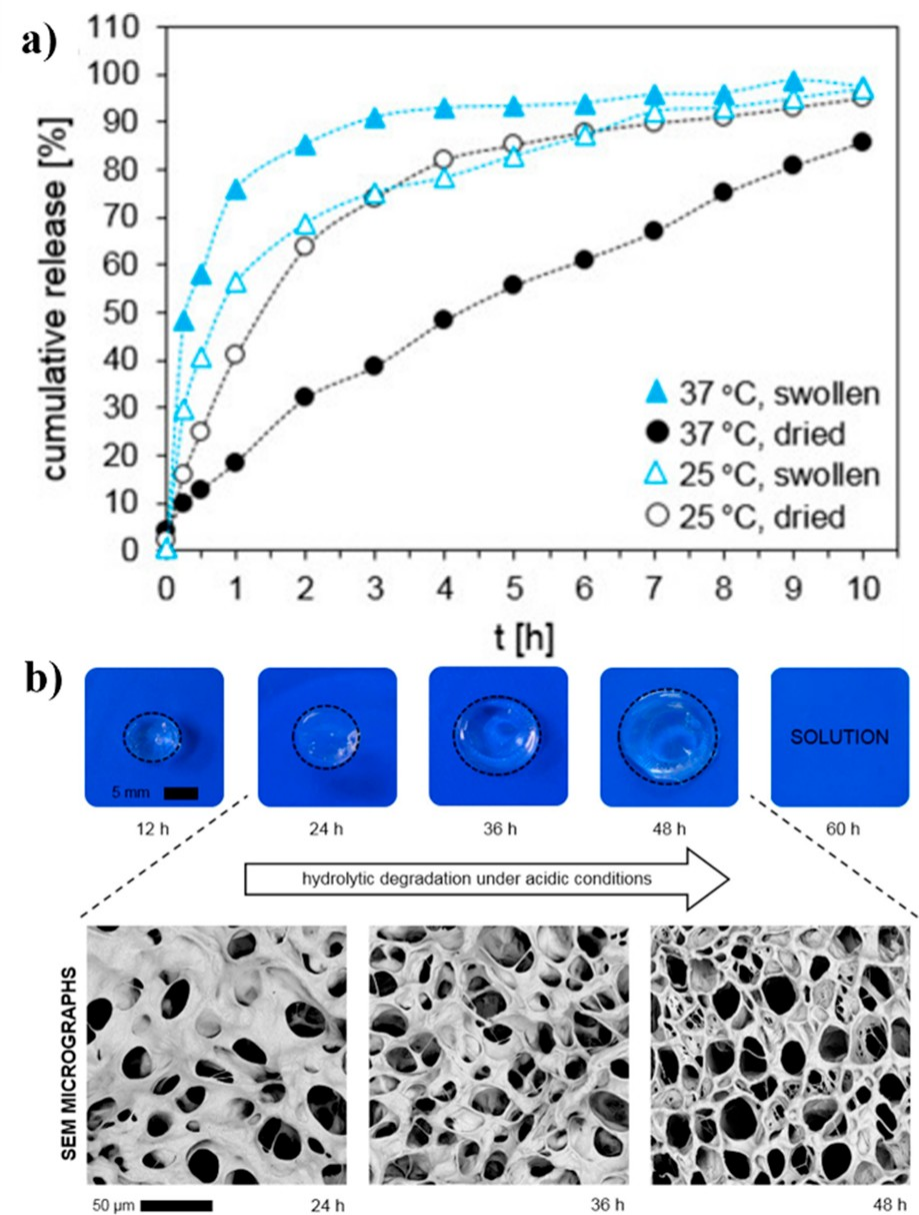
(a) Release profiles of BSA into PBS medium (pH = 7.4) from swollen and dried hydrogels at 37 and 25 °C. (b) Physical and morphological (SEM) appearance of a hydrogel during ongoing hydrolytic degradation in PBS pH 5.0 at 37 °C. Reproduced with permission from refs (95) and (94). Copyright 2017 and 2019, respectively, Elsevier.

In an effort to expand the functionality of trehalose-based hydrogels, Burek et al. incorporated NIPAM, mono- and bis-functionalized trehalose, and other hydrophilic comonomers into thermoresponsive diacetal trehalose hydrogels.^95^ By modifying the solvent system and the concentration of different components, these hydrogels could be tuned for specific LCST, VPTT, and degradation rates. Interestingly, model protein bovine serum albumin (BSA) was encapsulated in the hydrogels and the release rate could be controlled based on the temperature and swollen/dry state. That was particularly important to avoid the so-called “burst release”, that is, the sudden and immediate release of a high percentage of a drug payload upon administration. Typically, linear or sustained release is preferred to achieve a longer therapeutic effect, while controlled release is employed when time or stimuli responsiveness is desired. However, burst release can be utilized effectively for emergency treatments, where a high amount of drug needs to enter circulation rapidly. From swollen hydrogels, BSA was released in PBS pH 7.4 at 37 °C in a burst fashion, with about 60–70% of the protein released within 30 min. Nearly quantitative release was reached within 2–6 h regardless of the hydrogel components. However, if the gels were dried first, release was delayed with higher trehalose content hydrogels and proceeded in linear fashion, only reaching complete release in 5–8 h (Figure 20a). The hydrogels at different concentrations were also shown to stabilize β-Gal against acidic pH (pH 3.0) and 37 °C for 1.5–6 h with 65–95% (10 wt %) or 56–88% (5 wt %) enzyme activity remaining compared to 42–82% for β-gal alone.^95^ The authors further explored the tunability of the hydrogel degradability and subsequent protein release by using different trehalose diacetal cross-linkers that could be cleaved in acidic conditions and lead to hydrogel degradation, as demonstrated by SEM (Figure 20b), to yield soluble polymer chains and free trehalose.^94^ With this design, the release profile of BSA in PBS pH 5.0 at 37 °C was initially linear and sustained until degradation of the hydrogel and enlarging pore size resulted in a burst of BSA, typically after 40–50% had already been released. The onset of this degradation-based burst release of BSA at pH 5.0 began at 21–72 h with complete release in 30–100 h, but this burst was suppressed at the physiological pH of 7.4.^94^ This pH-dependent release profile could be useful in the treatment of diseases involving acidic environments, such as cancer.

**Figure 21:**
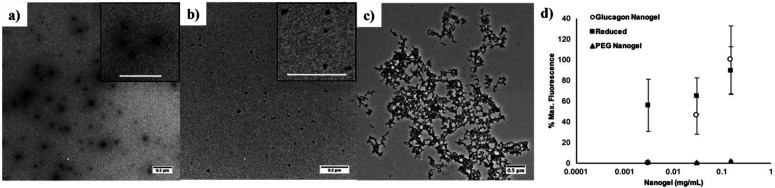
TEM images of glucagon nanogels in HEPES buffer at (a) day 7, (b) day 21, and (c) 3 days after TECP reduction. (d) Dose–response curves of glucagon nanogel before and after reduction, and PEG nanogel using Chem-1 cells expressing human glucagon receptor. Reproduced with permission from ref (58). Copyright 2018 Wiley.

Our group further explored responsive trehalose hydrogels by incorporating boronic acid into networks to confer glucose responsiveness for insulin release.^66^ By mixing pendant trehalose polymers with 8-arm PEGs end-capped with boronic acid groups, insulin was encapsulated within a hydrogel formed by the dynamic covalent bonds between boronic acid and trehalose. In a hyperglycemic event, the network is dissolved by glucose displacing trehalose in the boronic acid interaction, due to the 5.4× higher binding affinity of glucose with boronic acid, and the insulin released. The trehalose hydrogel stabilized insulin against accelerated heating conditions (90 °C, 30 min) with 74% intact insulin relative to 39% with the 8-arm PEG or only 2% with insulin alone. Furthermore, insulin was completely released from the hydrogel in 1 h at 1000 mg/dL glucose or 2 h at 500 mg/dL glucose, whereas at 0 mg/dL glucose only 60% of insulin was released in 2 h, demonstrating glucose-dependent release.^66^ Nonetheless, background release at 0 mg/dL was high, and thus, further tuning of the system would be needed to achieve useful on-demand insulin release. In a similar vein, glucagon, a highly unstable peptide used in hypoglycemia treatment, was entrapped within a nanosized trehalose network matrix to improve stabilization.^58^ Glucagon is a peptide notorious for its isoelectric point around physiological pH, making it very difficult to stably store in solution. Moreover, it can form toxic fibrils when in solution for a few days or exposed to higher temperatures. Glucagon was modified to have two free thiol groups and then used to cross-link the trehalose-PDSMA copolymer. The resulting nanogels increased the solution stability of glucagon from less than 24 h at physiological pH to at least 3 weeks. This was demonstrated by the lack of glucagon fibrils in transmission electron microscopy (TEM) images after 7 and 21 days in solution and by the appearance of the fibrils after reduction of the nanogel (Figure 21a–c). The in vitro activity of the thiolated glucagon was found to be similar to that of native glucagon. Glucagon released under mild reducing conditions was fully active (Figure 21d).^58^ Wang et al. prepared SENs for the stabilization of GOx using statistical or block copolymers. The former was able to generate stronger binding with the enzyme as shown by isothermal titration calorimetry (ITC), although DLS suggested that block copolymers favored the formation of SENs over multienzyme nanoparticles. Moreover, block copolymers were slightly better protein stabilizers (*T*_m_: 74.1–75.2 °C vs 72.7–73.2 °C), as measured by differential scanning calorimetry (DSC). A more pronounced stabilization effect was found when SENs were cross-linked (*T*_m_: 82.8–83.2 °C), indicating that physical entrapment is more important for this particular system than the nature of the polymer or the sugar. Increasing the percentage of trehalose to 100% in the cross-linked shell resulted in a higher *T*_m_ of 92.8 °C. The same trends could be found when measuring enzyme activity after exposure to 60 °C for 60 min, with remaining activity increasing from 25% to 65% after cross-linking and to 85% when encapsulated within a trehalose shell.^74^

Other recent examples of trehalose-based hydrogels have shown expanded uses for skin burn treatment^105^ and for cryopreservation and to act as a cell scaffold.^22,106^ Recently, microgels were used as soft matrices for 3D cell culture^99^ or in microfluidic microchambers.^107^

As with other hydrogel drug delivery systems, these trehalose-containing hydrogels and nanogels offer tunable release and degradation delivery matrices. Even the multifunctionalized trehalose used as monomers and cross-linkers still provide stabilization for the encapsulated proteins. Avoiding burst release is an ongoing issue, but the tunability on display already indicates that optimization is possible to match a specific application. Additionally, both thermoresponsive and chemically responsive trehalose hydrogels have already shown great promise for stabilization and controlled delivery of proteins.

### 3.2 Gene Delivery

Gene therapy recently witnessed a surge in translation to the clinic with the approval and widespread use of mRNA-based COVID-19 vaccines, and many more nanoparticle gene formulations for a range of diseases are currently in clinical trials.^108^ Polymeric materials play an important role in these nonviral gene delivery strategies, including trehalose materials.^109^

**Figure 22:**
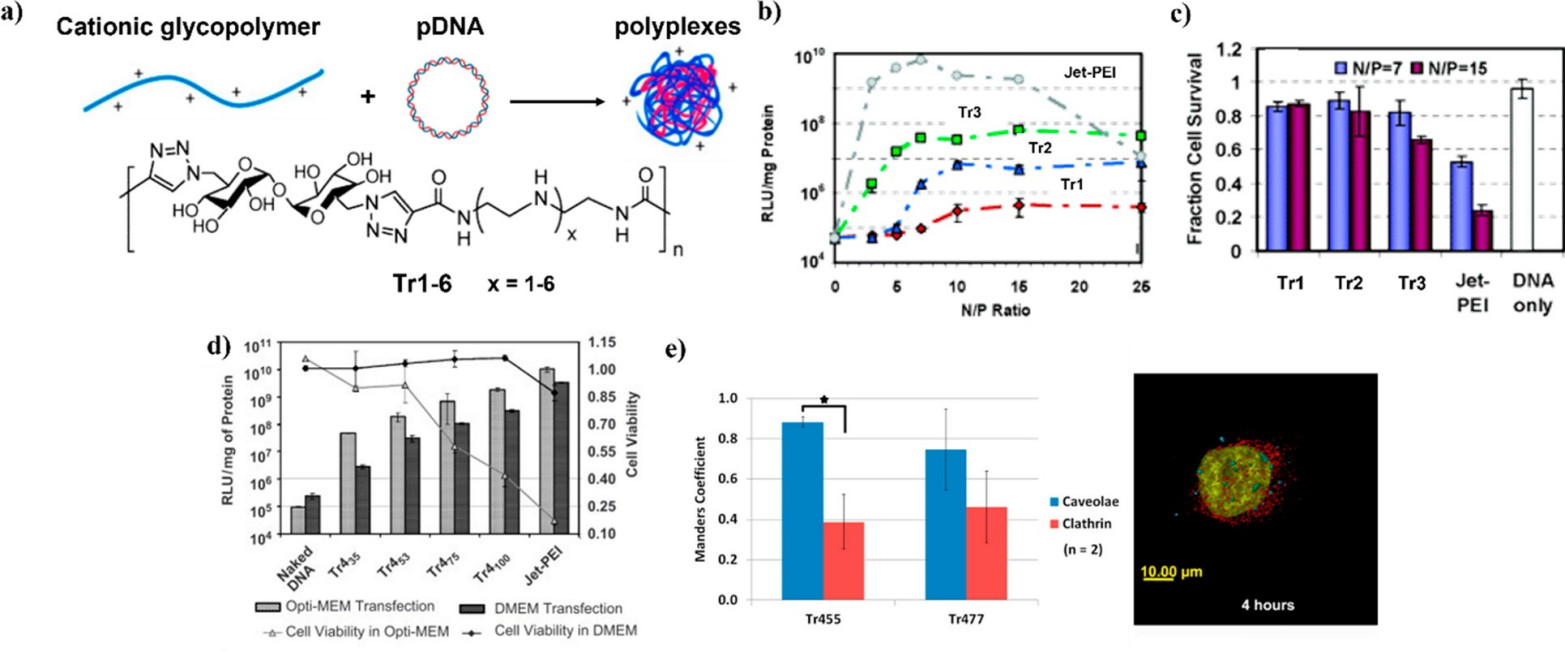
(a) Schematic representation of Tr1–6 and polyplex formation after complexation with pDNA. (b) Luciferase reporter gene expression in DMEM containing 10% serum. (c) Fraction of cell survival in DMEM containing 10% serum. Adapted from ref (42). Copyright 2006 American Chemical Society. (d) Optimum luciferase gene expression RLU/mg (bars) and the fraction of cell survival at optimum gene expression (lines) with HeLa cells. Reproduced with permission from ref (43). Copyright 2007 Elsevier. (e) Manders coefficient for colocalization of polyplexes with clathrin and caveolae and colocalization of polyplexes with Rab 5 proteins at 4 h for Tr455 showing perinuclear localization of polyplexes. Reproduced from ref (47). Copyright 2013 American Chemical Society.

Backbone poly(trehalose) was selected early on by the Reineke group to prepare cationic copolymers carrying amidines^38^ or quaternary amines^39^ for plasmid DNA (pDNA) stabilization and delivery. The influence of sugar size, charge spacing, and charge type of the trehalose polymer polyplexes on transfection, toxicity, and pDNA stabilization were investigated. Amidine-based polyplexes demonstrated higher transfection ability than quaternary amines while achieving comparable toxicity.^39^ The presence of trehalose significantly lowered the cytotoxicity.^38^ This work was expanded using a click-chemistry-based synthetic strategy to prepare cationic glycopolymers with trehalose in the backbone as described above.^42−51^ While trehalose promoted stability and prevented aggregation, the cationic units interacted with DNA phosphate groups and amido-triazole units promoted DNA binding via hydrogen bonding and hydrophobic interactions. As such, triazole containing polymers complexed pDNA at lower amine/phosphate (N/P) ratios when compared to analogues without triazoles. Moreover, increasing the number of amine repeating units (1–6, polymers labeled Tr1–Tr6, Figure 22a) resulted in higher pDNA affinity, polyplex stability in cell media, pDNA transfection, and gene expression in HeLa cells.^42^ Nonetheless, although cellular uptake was higher than that for Jet-PEI, a common polymer used for gene delivery, gene expression was lower, possibly indicating low endosomal escape (Figure 22b). Higher amine content yielded higher cell toxicity, but trehalose copolymers were still much less toxic than Jet-PEI (70% vs 25% at N/P = 15) (Figure 22c).^42^ While Tr1 was found to interact with pDNA through an electrostatic mechanism, Tr3 and especially Tr4 were more dependent on base pair interactions though hydrogen bonding, probably due to the longer spacer between amine groups.^44^

Although the amine number did initially have a significant effect on polyplex formation, transfection, and stability, the effect tailed off at higher numbers (Tr5, Tr6), as evidenced by reduced transfection of polyplexed pDNA into rat mesenchymal stem cells (RMSC) (20% Tr4 vs 10% Tr5 vs 8% Tr6).^46^ To investigate chain length effect on biological properties, Tr4 was prepared at different DP (35, 53, 75, and 100). Interestingly, while chain length had no apparent impact on pDNA binding, heparin displacement, ITC, pDNA degradation, or gene uptake, increasing the DP resulted in higher polyplex stability in complete media and higher gene expression, but with an unfortunate increase of toxicity in HeLa cells (Figure 22d).^43^ Moreover, higher DP Tr4 showed an impressive 40% transfection of pDNA in RMSCs,^46^ demonstrating that as expected transfection is dependent on cell type. Further, exploring different polymer end groups of Tr4 polymers led to the discovery that carboxyl, octyl, and oligoethyleneamine groups caused higher pDNA uptake and gene expression than other end groups, including adamantane, alkynyl-oligoethyleneamine, and azido trehalose in HeLa cells.^45^ The azido-trehalose end-capped Tr4 was also found to have reduced efficacy compared to PEGs and triphenylacetamide end groups in RMSCs.^46^ Additionally, 4D spatiotemporal cellular imaging was used to demonstrate that decreasing nanoparticle size allowed for faster advancement to the perinuclear zone. In particular, Tr4 was internalized via the caveolae/Rab-5 dependent pathway, a type of endocytosis involving the formation of flask-shaped vesicles of the cell plasma membrane, and reached the area within 4 h of cellular internalization (Figure 22e).^47^

**Figure 23:**
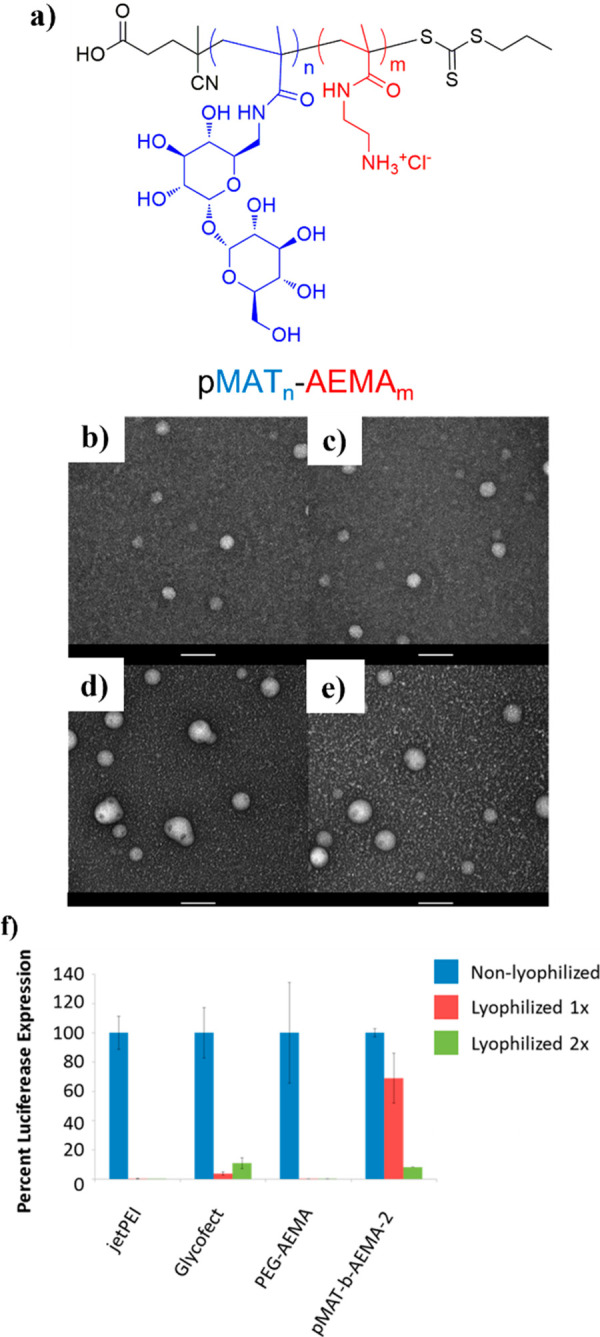
(a) Schematic representation of RAFT side chain trehalose-cation block copolymers. (b–e) TEM images of p(trehalose-*b*-cation) with increasing cation block MW (a,d: DP = 21; b,e: DP = 44), (b, d) fresh polyplexes and (c, e) after lyophilization and reconstitution. Scale bar: 100 nm. (f) Luciferase expression in U87 cells following transfection with lyophilized polyplexes (AEMA: cationic block; pMAT: trehalose block). Adapted from ref (110). Copyright 2015 American Chemical Society.

Importantly, RAFT side chain trehalose-cation block copolymers stabilized pDNA polyplexes against one cycle of lyophilization and reconstitution; both colloidal stability and gene delivery ability were retained after the physical process, outperforming PEG analogues (Figure 23).^110^ Other than pDNA, Tr4^48,49^ and RAFT^71^ copolymers were used to deliver small interfering RNA (siRNA), demonstrating theragnostic abilities^49^ and colloidal and freeze/drying stabilization properties.^71^

Finally, additives to increase transfection efficiencies and protein expression have been explored for delivering pDNA with trehalose-based polymers. Increasing the heparin concentration was found to linearly increase green fluorescent protein (GFP) expression in primary fibroblasts (PFB), human liver carcinoma HepG2, and human glioblastoma U87 cells (4-fold), despite only increasing cellular internalization in HepG2 cells. It is possible that this discrepancy is due to heparin-coated Tr4 polyplexes being taken up by a different endocytic pathway (clathrin-mediated endocytosis and micropinocytosis) or to enhanced nuclear delivery.^50^ The effect of plasmid size on gene delivery was studied with heparin-coated polyplexes, and, predictably, larger plasmids (10 kbp) had reduced gene expression in primary human dermal fibroblasts (HDFs) and induced pluripotent stem cells (iPSCs). An alternative additive, dexamethasone, was used to destabilize the nuclear barrier and increase trafficking enhancing transfection and expression.^51^

Both backbone and side chain trehalose polymers were used to deliver genetic material to cells and increase protein expression. Most studies focused on pDNA transfection, and overall Tr4 emerged as the best candidate in term of uptake, gene expression and low toxicity. However, biological properties can be further tuned by changing chemical parameters such as DP or chain end functionalities. Interestingly, the use of RAFT polymerization allowed the preparation of block copolymers, with trehalose and cationic block distinctly separated, whereas click chemistry afforded alternate copolymers with each unit of trehalose separated by a cationic monomer. Although a direct comparison of the two strategies has not been reported yet, it is not surprising that RAFT block copolymers were able to stabilize polyplexes to lyophilization cycles, likely due to the greater clustering effect of the trehalose block, and even deliver pDNA in vivo. Conversely, Tr1–6 alternating backbone copolymers formed more stable polyplexes in complex media, possibly due to the higher interaction and stabilization provided by the triazole moiety, which results also in high uptake and transfection.

### 3.3 Aggregate Prevention for Amyloid Disorders

**Figure 24:**
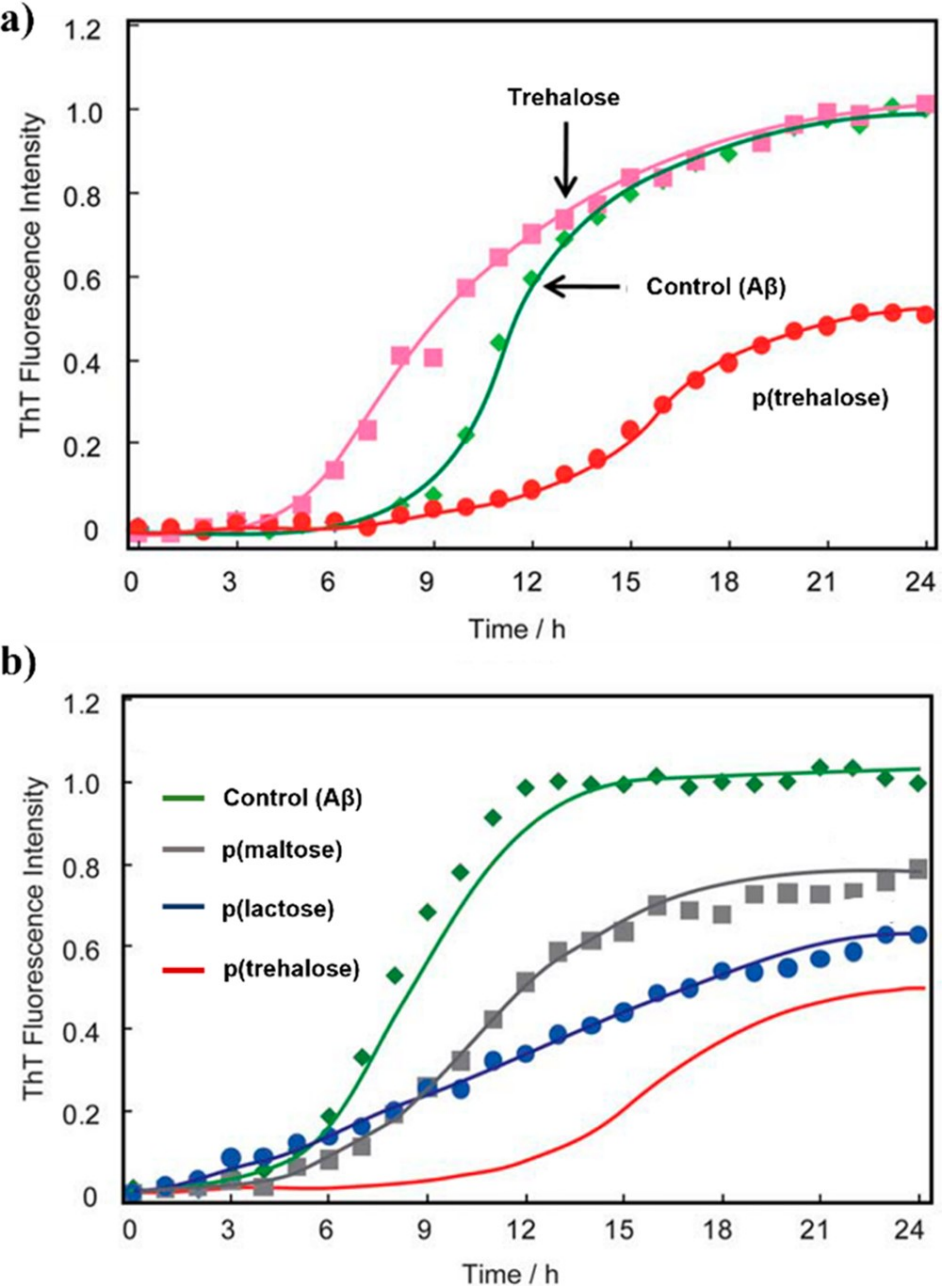
Thioflavin T assay for Aβ fibril detection over time at pH 7.4, 37 °C. (a) Green: no additives; pink: molecular trehalose; red: p(trehalose). (b) Green: no additives; gray: p(maltose); blue: p(lactose); red: p(trehalose). Adapted from ref (57) with permission from the Royal Society of Chemistry.

Trehalose has been found to be effective in the treatment of different neurodegenerative pathologies including Alzheimer’s, Parkinson’s, and Huntington’s diseases.^111,112^ Although the exact mechanism is not clear yet, it likely includes antiaggregation, anti-inflammation, and, in particular, autophagy induction, that is, the intracellular removal or destruction of unnecessary or dysfunctional components.^113^ Trehalose glycoclusters^114^ and nanocarriers^23^ were more efficient in delaying fibril formation and protein aggregation and protecting neurons than the small molecule trehalose, indicating that the cluster effect seen in stabilizing proteins with trehalose polymers is also applicable. Recently, poly(trehalose) was also found to be effective in preventing Aβ peptide aggregation, part of the progression of Alzheimer’s disease. In two instances, Miura and co-workers prepared poly(trehalose)s by FRP and studied their effect on Aβ aggregation inhibition.^56,57^ The polymer with a short adipoyl trehalose spacer showed greater aggregation inhibition than molecular trehalose, 20% vs 60% aggregation, respectively, protecting HeLa cells from Aβ cytotoxicity. However, a longer alkyl side spacer, sebacoyl, was found to induce aggregation.^56^ To eliminate the spacer contribution, acrylamide-trehalose copolymers were prepared and found to better prevent Aβ aggregation than small molecule trehalose-, maltose-, and lactose-based polymers, again decreasing Aβ aggregate cytotoxicity (Figure 24). Surprisingly, polymers with higher trehalose content were not more efficient at preventing aggregation, possibly due to higher steric hindrance and difficulties generating hydrogen bonds.^57^

Notably, Debnath et al. prepared poly(trehalose) zwitterionic nanoparticles with an iron oxide core that were able to bind and completely disintegrate mature Aβ peptide or HEWL fibrils in 10 days or 20 h, respectively. These rates were 3–4 orders of magnitude more effective than that of molecular trehalose in preventing aggregation and aggregation-derived cytotoxicity in a Huntington’s disease cell or mouse model.^115^ The same group found similar results for other neurodegenerative diseases using different trehalose nanoparticles: with a gold core,^116^ dendrimer-based,^117^ poly(lactide)-based to confer degradability and biocompatibility,^118^ or prepared by heating/carbonization of trehalose.^21^ In every case, the nanoparticles were more effective than molecular trehalose, again indicating that trehalose is more effective in a multivalent system than in small molecule form.

### 3.4 Others

**Figure 25:**
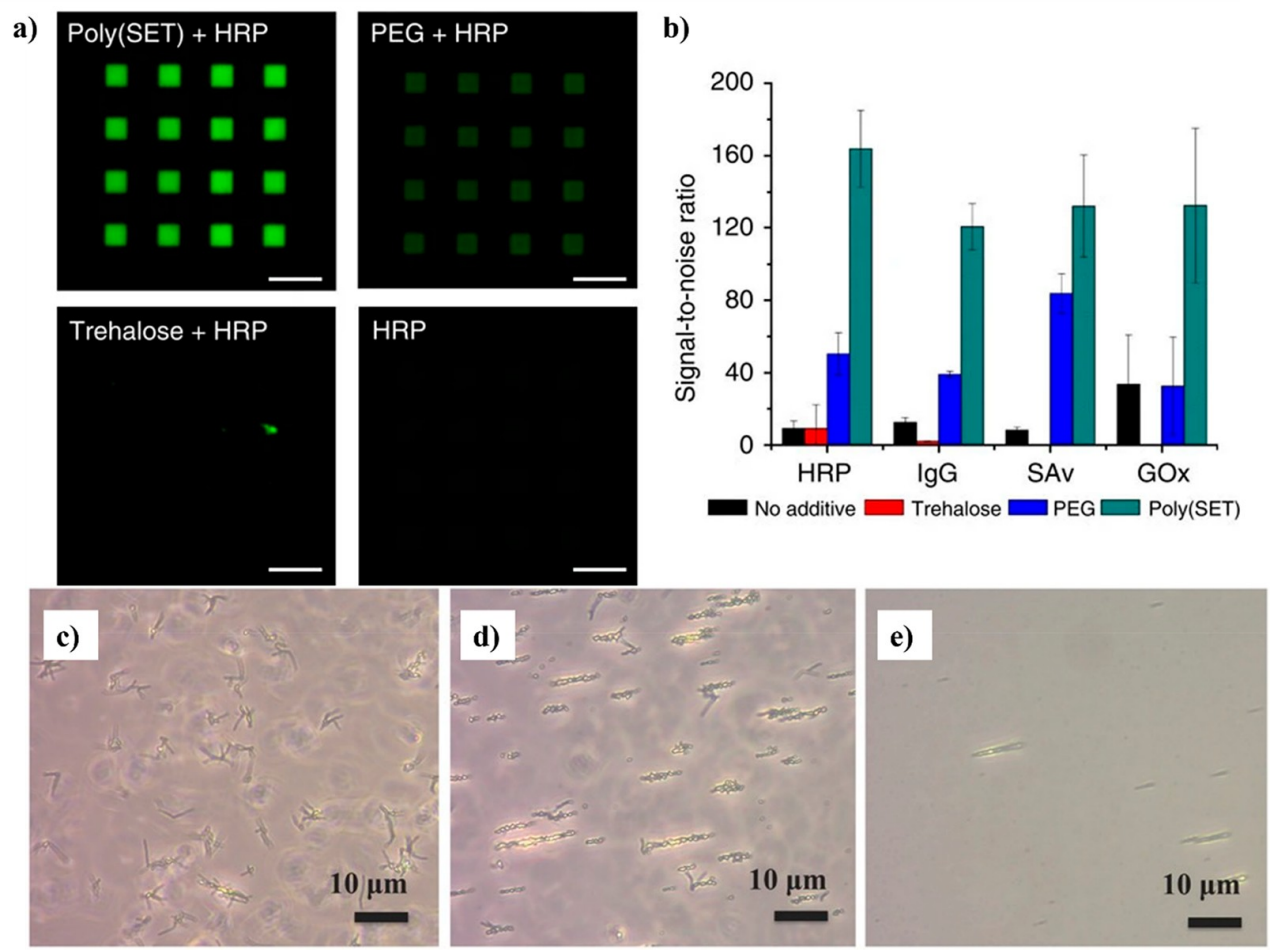
(a) Fluorescence micrographs of HRP patterned with poly(SET), PEG, trehalose or without an additive after staining with AlexaFluor 488 goat anti-HRP. Scale bars, 25 μm. (b) Signal to noise ratios calculated for different proteins patterned with different excipients. Reproduced with permission from ref (119). Copyright 2015 Springer Nature. (c–e) Optical images of magnetic micelles treated with *M. smegmatis* mc2 155 (108 CFU mL^–1^) (c) before and (d) after placing a magnet to the left of the sample. (e) *M. smegmatis* mc2 155 (104 CFU mL^–1^) after applying a magnet. Reproduced with permission from ref (121). Copyright 2016 Wiley

Beyond the applications discussed thus far, there have been assorted forays into other realms including photolithographic printing of proteins and metal-incorporated nanoparticles for the detection of bacteria and infection prevention. Trehalose polymers have enabled the direct-write electron-beam lithography of multiple proteins into nanometric and submicrometer width lines and patterns. Direct write of proteins is not possible because the process requires harsh electron beam irradiation and vacuum. By spin coating proteins with a styrenyl ether trehalose polymer (polySET), many proteins were able to withstand multiple iterations of these harsh conditions. The original experiments patterning HRP, GOx, IgG (chicken, human, mouse), streptavidin (SAv), vascular endothelial growth factor (VEGF), and basic fibroblast growth factor (bFGF) found that there was significantly more active protein with the trehalose polymer than with trehalose, PEG, or nothing (Figure 25a,b).^119^ This protein patterning method was applied as the basis for a sandwich immunoassay measuring inflammatory cytokine production by localized surface plasmon resonance (LSPR).^120^ Antibodies for two common cytokines, interleukin-6 (IL-6), and tumor necrosis factor-α (TNF-α) could be sequentially lithographically patterned using the trehalose polymer and still capture their respective cytokine from LPS-stimulated cell media.

In the pursuit of detecting and treating bacterial infections, trehalose polymers have been incorporated into nanoparticles encapsulating iron oxide,^121^ poly(trehalose acrylate) AuNPs,^69^ or CNFs.^70^ As trehalose is a key component of the cell wall and glycolipids of some organisms, some bacteria, including mycobacteria, have lectins that will specifically bind to trehalose. Based on an earlier iteration of iron oxide nanoparticles directly incorporating trehalose that interacted strongly with *Mycobacterium smegmatis*,^122^ researchers synthesized iron oxide encapsulated pendant trehalose poly(lactic acid) polymer micelle nanoparticles.^121^ The nanoparticles not only specifically detected *M. smegmatis* over *Staphylococcus epidermidis* and *Escherichia coli* (Gram-positive and Gram-negative, respectively) but also could facilitate bacteria removal by magnetically dragging the trehalose-lectin bound bacteria (Figure 25c–e). Using similar trehalose–lectin interactions, it was possible to reduce bacterial infection by grafting a RAFT polymerized poly(trehalose acrylate) to AuNPs by thiol–gold interaction^69^ or to CNFs using aldehyde-functionalized poly(trehalose) via Passerini reaction.^70^ The trehalose nanoparticles prevented HUVECs from being infected with *Staphylococcus aureus* by preferentially binding with HUVEC lectins, thereby inhibiting the first step in bacterial infection, i.e., adhesion via lectin-glycan bonding. Trehalose-coated AuNPs and CNFs were found to be noncytotoxic up to 0.75 and 0.50 mg/mL, respectively, for either mouse macrophage RAW 264.7 cells or HUVECs.

## 4 Outlook and Future Perspective

In terms of academic research, trehalose has long been used as a stabilizer for a variety of biologics, such as protein and genetic material, viruses, and microorganisms,^123^ or as a cryoprotectant for cells and tissues.^124^ Moreover, trehalose is being explored as a therapeutic for cancer^125^ and metabolic diseases,^126^ as an autophagy inducer,^127^ and as a stabilizer for nebulized formulations that does not induce toxicity in the lungs,^128^ among many other applications. However, in the past decade, poly(trehalose) has emerged as a promising and potentially superior alternative to the sugar alone. In this Perspective, recent advances in the synthesis and applications of trehalose-based linear polymers, resins, gels, and nanoparticles were presented and discussed. Based on the presented knowledge, some open questions remain: How do polymers with trehalose in the backbone or in the side chain compare against each other? How can the synthesis be improved to increase yields and avoid tedious protection steps? What is the in vivo safety and metabolic fate of trehalose polymers? Which other application or diseases can be tackled with poly(trehalose)? More generally, what does the future hold for this sugar with unique properties?

The Maynard group has pioneered the use of poly(trehalose) for protein stabilization, and similarly the Reineke group has advanced the field for gene therapy with many contributions. Nonetheless, despite the widespread and successful use of poly(trehalose) for biologic stabilization, there is a surprising lack of research into the use of poly(trehalose) as a therapeutic. Only a few examples can be found in the literature, including work from Miura and co-workers that first demonstrated the efficacy of poly(trehalose) in the treatment of neurodegenerative disease. In more recent years, the Jana group proposed many formulation strategies to advance this research field. Additionally, in the last couple of years, the Langer and Stenzel groups simultaneously proved how poly(trehalose) is effective in the detection and prevention of bacterial infections. Considering the wide variety of therapeutic applications that have been explored for molecular trehalose, we expect that poly(trehalose) may similarly provide more therapeutic opportunities yet to be developed.

Similarly, the success of trehalose in the pharmaceutical industry may indicate the great potential of poly(trehalose) use in the future. Specifically, in 2011, there were only four U.S. Food and Drug Administration (FDA) approved therapeutic formulations (Herceptin, Avastin, Lucentis, and Advante) containing trehalose as an excipient, while several others were approved in Japan. Moreover, trehalose was already widely used in the food and cosmetic industries^123^ and was classified as GRAS (generally regarded as safe) by the FDA. Just a decade later, with the increased use and technology advancement of protein therapeutics, trehalose usage has witnessed an incredible surge in the biopharmaceutical field and in FDA approved products. In the antibody production field alone, trehalose is now the second most commonly used sugar for osmolality-adjustment, with over 20 antibody formulations, and it is the second most common sugar used as a lyoprotectant, present in 11 products.^129^ Furthermore, the biocompatibility of trehalose makes it an ideal candidate for other fields, including plastic manufacturing and agriculture. In this regard, some of the trehalose-based thermoset resins already showed their potential. One could assert that molecular trehalose is fully explored for its potential. Considering its similarity to trehalose, the next step in the field should be to further explore poly(trehalose) as an excipient for therapeutic applications. A multitude of trehalose polymers have already shown increased efficacy in stabilizing and preventing biomacromolecules aggregation compared to molecular trehalose and other sugars both in vitro and in vivo, making poly(trehalose) an interesting candidate for incorporation into therapeutic protein formulations. Many synthetic strategies have proven successful in synthesizing trehalose polymers, with click chemistry and controlled radical polymerization being the most popular preparation methods for backbone and side chain trehalose polymers, respectively. Nonetheless, monomer preparation is still challenging, often requiring tedious protection/deprotection steps and providing low yields. An improved synthetic methodology is required to move poly(trehalose) toward the clinic. Further, despite all this promising research, few studies on the safety and in vivo fate of trehalose polymers can be found. Comprehensive studies regarding poly(trehalose) biodistribution, long-term accumulation, pharmacokinetics, immunogenicity, and toxicity in general are needed to advance this polymer that shows great potential for routine use in everyday life.
